# Investigating the beneficial traits of *Trichoderma hamatum* GD12 for sustainable agriculture—insights from genomics

**DOI:** 10.3389/fpls.2013.00258

**Published:** 2013-07-30

**Authors:** David J. Studholme, Beverley Harris, Kate Le Cocq, Rebecca Winsbury, Venura Perera, Lauren Ryder, Jane L. Ward, Michael H. Beale, Chris R. Thornton, Murray Grant

**Affiliations:** ^1^Biosciences, Molecular Plant Pathology, College of Life and Environmental Sciences, University of ExeterExeter, UK; ^2^Plant Biology and Crop Science, Rothamsted ResearchHarpenden, UK

**Keywords:** *Trichoderma hamatum*, comparative genomics, secretome, plant growth promotion, induced systemic resistance

## Abstract

*Trichoderma hamatum* strain GD12 is unique in that it can promote plant growth, activate biocontrol against pre- and post-emergence soil pathogens and can induce systemic resistance to foliar pathogens. This study extends previous work in lettuce to demonstrate that GD12 can confer beneficial agronomic traits to other plants, providing examples of plant growth promotion in the model dicot, *Arabidopsis thaliana* and induced foliar resistance to *Magnaporthe oryzae* in the model monocot rice. We further characterize the lettuce-*T. hamatum* interaction to show that bran extracts from GD12 and an *N-acetyl-β-D-glucosamindase-deficient* mutant differentially promote growth in a concentration dependent manner, and these differences correlate with differences in the small molecule secretome. We show that GD12 mycoparasitises a range of isolates of the pre-emergence soil pathogen *Sclerotinia sclerotiorum* and that this interaction induces a further increase in plant growth promotion above that conferred by GD12. To understand the genetic potential encoded by *T. hamatum* GD12 and to facilitate its use as a model beneficial organism to study plant growth promotion, induced systemic resistance and mycoparasitism we present *de novo* genome sequence data. We compare GD12 with other published *Trichoderma* genomes and show that *T. hamatum* GD12 contains unique genomic regions with the potential to encode novel bioactive metabolites that may contribute to GD12's agrochemically important traits.

## Introduction

With the global population estimated to reach 9 billion by 2050, current plant breeding approaches alone will not support the increased demand for food. There is an urgent need to investigate alternative, sustainable approaches to enhance agricultural production. Additional pressures on food production such as existing and emerging pathogens (Anderson et al., 2004; Fisher et al., 2012), soil erosion (Montgomery, 2007), reduced water and nutrient availability (Sauer et al., 2010; Powlson et al., 2011), climate change (Schmidhuber and Tubiello, 2007) and competition for available land from other sectors such as house building and biofuels (Harvey and Pilgrim, 2011), will add further pressure on agricultural systems to maximize crop productivity. Moreover, hazard-based criteria for assessing pesticides could lead to a range of agrochemicals being withdrawn from European markets, leading to the potential loss of the only effective fungicide groups against major crop diseases.

These challenges have lead to research into alternative sustainable agricultural strategies, with a strong focus on exploiting beneficial organisms. Members of the fungal genus *Trichoderma* have the potential for reducing existing dependence on the use of environmentally damaging and unsustainable chemicals required for disease control and fertilizers (Fantke et al., 2012), by providing an opportunity to sustainably improve crop productivity while reducing the likelihood of development of fungicide resistant pathogens.

*Trichoderma* is a member of the Ascomycota, the largest group of fungi. Asexual reproduction occurs through the production and germination of asexual conidia (Steyaert et al., 2010) and in some species of *Trichoderma*, sexual teleomorphic stages (*Hypocrea* spp.) have been identified (Seidl et al., 2009), although *Trichoderma* is now the accepted holomorph nomenclature (International Botanical Congress, 2011). *Trichoderma* has been exploited in many industries including paper, textile, biofuel and agriculture due to its prolific secretion of degrading enzymes and biocontrol activities (Pere et al., 2001; Miettinen-Oinonen and Suominen, 2002; Chaverri et al., 2003; Giraldo et al., 2007; Kuhad et al., 2011).

Biocontrol encompasses a variety of mechanisms working singularly or synergistically during the interaction between a biological control agent, plant pathogen and plant to achieve effective disease control (Howell, 2003). These mechanisms can be either indirect, via competition for nutrients and space, antibiosis and stimulation of plant-defense mechanisms or direct mycoparasitism, or they can be a combination of both. Mycoparasitism involves direct antagonism of soil-borne pathogens by a combination of enzymatic lysis through secretion of chitinases, glucanases, proteases, antibiotic production, and competition for space and substrates (Harman, 2006; Lorito et al., 2010). Since the 1930's, *Trichoderma'*s mycoparasitic biocontrol activities have been extensively used in agriculture. Research has focussed predominately on *Trichoderma virens, T. atroviride, T. asperelloides*, *T. asperellum* and *T. harzianum* (Howell, 2003; Benitez et al., 2004). However, mycoparasitism is widespread. More than 1100 *Trichoderma* strains from 75 molecularly defined species displayed mycoparasitic potential against the pathogens *Alternaria alternata, Botrytis cinerea* and *Sclerotinia sclerotiorum* [reported in Druzhinina et al. (2011)]. Yet, despite its agronomic importance, our current knowledge about the mechanistic basis for mycoparasitism is rudimentary.

Certain *Trichoderma* strains have been shown to stimulate plant growth through the production of plant-growth-promoting (PGP) compounds (Chang et al., 1986; Ousley et al., 1994; Contreras-Cornejo et al., 2009; Vinale et al., 2009) although both biological control and PGP traits are rarely found together. Often, PGP is unpredictable and is influenced by environmental factors (Maplestone et al., 1991; Ousley et al., 1993). The mechanisms for PGP are thought to variously arise from direct effects on plants, decreased activity of microflora and inactivated toxic compounds in the root zone (Harman et al., 2004). *Trichoderma* species can also ameliorate a wide range of abiotic stresses such as salinity, temperature and drought; they can improve photosynthetic efficiency, enhance nutrient uptake and significantly increase nitrogen use efficiency in crops. These are all attributes that can contribute to enhanced PGP characteristics often evident upon inoculation (Harman et al., 2004; Djonovic et al., 2006; Bae et al., 2009; Shoresh et al., 2010). Strains stimulate PGP through the production of, yet to be defined, PG compounds (Contreras-Cornejo et al., 2009; Vinale et al., 2009; Ryder et al., 2012), most likely through a combination of one or more of the remarkably diverse array of secondary metabolites and proteins such as pyrones, peptaibols, and terpenes (Lorito et al., 2010) that *Trichoderma* produces.

In addition to mycoparasitism and PGP, some *Trichoderma* strains can induce broad spectrum systemic resistance (ISR) in leaves (Shoresh et al., 2010). Generally it is accepted that, in agricultural systems, the activation of defense responses generates a “trade-off” in terms of reduced growth or enhanced susceptibility to other stresses (Heidel et al., 2004; van Hulten et al., 2006). Remarkably, however, *Trichoderma* inoculation can ameliorate these traditionally perceived “costs” suggesting that it can locally suppress MAMP (Microbe Associated Molecular Pattern) triggered immunity (MTI) and systemically activate or prime induced plant immunity. Suppression of MTI has been recently demonstrated for the plant growth promoting rhizobacterium (PGPR) *Pseudomonas fluorescens* strain WCS417r, which grows endophytically or on root surfaces (Millet et al., 2010).

Modifications of hormonal balance by host or microbe are key drivers in determining the outcome of plant-pathogen interactions, including suppression of MTI (Grant and Jones, 2009). ISR induced by *P. fluorescens* WCS417r is mediated through jasmonic acid/ethylene (JA/ET) signaling (Ton et al., 2002; Santner and Estelle, 2007). Evidence for engagement of specific hormone signaling in *Trichoderma* ISR is often contradictory, with various *Trichoderma* strains activating ISR through different signaling modules. The ISR mediated by *T. asperellum* T34 appears to parallel the JA/ET-based “priming” events observed for *P. fluorescens* WCS417r, resulting in enhanced resistance to obligate biotrophs, hemi-biotrophs and necrotrophs (Segarra et al., 2009). By contrast, maize inoculated with *T. harzianum* T22 showed constitutive expression of some PR proteins in the absence of a pathogen. In melon, *T. harzianum* can control *Fusarium* wilt through induction of basal resistance and the attenuation of hormonal disruption of abscisic acid (ABA), salicylic acid (SA) and ET signaling that *Fusarium oxysporum* induces (Martinez-Medina et al., 2010, 2011). Recently, ISR, induced by *T. hamatum* T382 against *Botrytis cinerea* in *A. thaliana* was reported to involve both an initial priming and a post-infection response (Mathys et al., 2012). Thus, current knowledge suggests that induction of ISR depends upon the specific strain. Host genotype also contributes since genetic variability between tomato lines determines the outcome of PGP and biocontrol interactions with *T. atroviride* and *T. harzianum* (Tucci et al., 2011).

*Trichoderma hamatum* is a naturally occurring rhizosphere dwelling member of the genus which has attracted academic and industrial interest due to its ability to increase plant biomass and its potential as a biological control agent (Chet et al., 1981; Elad, 2000; Harman, 2006). A previously described strain of *Trichoderma hamatum* (GD12) isolated from soil in Devon, UK, promotes plant growth in low pH, nutrient poor peat and displays biological protection against pre- and post-emergence diseases of lettuce seedlings caused, respectively, by *Sclerotinia sclerotiorum* and *Rhizoctonia solani*, under the same conditions (Thornton, 2005, 2008; Ryder et al., 2012). While plant growth promotion and biocontrol by *Trichoderma* have been well-documented, both traits rarely occur together (Contreras-Cornejo et al., 2009; Vinale et al., 2009). Whole genome sequences are becoming increasingly available, with the industrial strain *T. reesei*, and biological control strains *T. atroviride, T. harzianum*, *T. virens, T. longibrachiatum, T. citrinoviride, T. asperellum* now accessible in public repositories (http://tinyurl.com/trichoderma). Genome comparisons between the mycoparasitic *T. atroviride* and *T. virens* species vs. the saprophytic *T. reesei* identified components predicted to contribute to a parasitic lifestyle and a genome reduction in *T. reesei* (Kubicek et al., 2011). Availability of the genome sequence of GD12 would provide a valuable insight into the genetic potential underlying these important agronomic traits.

Here we present further characterization of the dual PGP and biocontrol strain *T. hamatum* GD12. To demonstrate the broad utility of GD12 as an experimental system we extend previous work to show (i) GD12 induced PGP of *Arabidopsis thaliana*, (ii) GD12 mycoparasitized isolates of the pre-emergent soil pathogen, *Sclerotinia sclerotiorum* and this antagonistic interaction resulted in further enhanced lettuce PGP, (iii) PGP of lettuce by sterile bran extracts from GD12 which is further enhanced by extracts from the GD12 *N*-acetyl-β-D-glucosaminidase deficiency mutant, (iv) clear differences in GD12 and the *N*-acetyl-β-D-glucosaminidase deficient mutant secretome fingerprint which may account for the difference in biocontrol and PGP and (v) induction of induced systemic resistance in rice to rice blast by both GD12 and the *N*-acetyl-β-D-glucosaminidase deficiency. To provide genomic resource to predict important components involved in PGP and biological control fitness of *T. hamatum* GD12 we undertook whole genome sequencing of this strain and compared it to sequenced *Trichoderma* strains (*T. atroviride. T. harzianum* and *T. virens).* This work revealed substantial differences between strains which allowed us to identify genomic regions/clusters unique to GD12 that can be further studied to gain a more comprehensive understanding of genetic basis for PGP, biocontrol against pre- and post-emergence soil pathogens and induced systemic resistance to foliar pathogens. In sum, this study provides a foundation for further dissection of GD12's ability to promote beneficial attributes.

## Materials and methods

### Plant growth promotion and biological control assays

#### Peat microcosms

One litre of sieved sphagnum moss peat (Shamrock, Scotts Professional, UK) was mixed with 400 ml dH_2_O and sterilized by autoclaving. Twenty-five lettuce seeds (*Lactuca sativa* cultivar Webb's Wonderful) were sown into triplicate 120 mm × 120 mm × 12 mm square plastic culture dishes (Greiner, Bio-One, UK) each containing 100 g sterile peat. For plant growth promotion and biological control assays, microcosms 300 g of peat was supplemented with: 8 g *T. hamatum* bran inoculum, *Sclerotinia* poppy seed inoculum or both (Ryder et al., 2012). Microcosms were maintained at 24°C under a 16 h light 8 h dark cycle at 90% humidity. Following the removal of lids after 48 h, microcosms were watered daily with sterile dH_2_O. After 21 days, plants were harvested, washed and oven dried (75°C) to a constant weight. Shoot and root fresh and dry weights were determined and the data analysed by using ANOVA and *t*-tests.

#### Bran extracts

Bran inoculum was prepared by inoculating a sterile bran mixture (250 ml conical flask containing 10 g wheat bran (Badminton Horse Feeds, UK) and 30 ml sterile dH_2_O with five 4 mm plugs of agar from the leading edge of a 3-day-old *T. hamatum* culture, grown on Potato Dextrose Agar (Sigma-Aldrich). The inoculum was incubated for 5 days at 26°C under a 16 h light regime.

#### S. sclerotiorum poppy seed inoculum

A sterile poppy seed mixture (250 ml conical flask containing 10 g black poppy seeds with 10 ml sterile dH_2_O) was inoculated with ten 1 mm plugs of agar from the leading edge of a 3-day-old *S. sclerotiorum* culture grown on PDA. The inoculum was incubated for 10 days at 26°C under a 16 h light regime. The four *S. sclerotiorum* isolates used in this study, BFS, GFR1, GFR11 and M488 were obtained from Dr Jon West, Rothamsted Research, Harpenden, UK.

### Rewatering assay

One Hundred microliters dH_2_O was added to triplicate 5 day old *Trichoderma hamatum* bran inoculum flasks (prepared as above) for each strain to be tested. Samples were mixed for 1 h and filtered through miracloth (Calbiochem) into 2 × 50 ml aliquots. Samples were centrifuged at 10,000 g for 10 min. and vacuum filtered through 5 μm filter paper (Whatman) and autoclaved for 15 min. at 121°C. Seedlings were watered with filtrate on alternate days for 21 days.

### Biocontrol assay

*Magnaporthe oryzae* leaf infection assays were carried out using dwarf Indica rice (*Oryza sativa* cultivar CO-39, which is susceptible to rice blast. Eight seedlings of CO-39 were planted in 15 pots (7 cm) and grown for 14 days (2–3 leaf stage) in soil containing *Trichoderma*-bran inoculum prior to *M. oryzae* strain Guy-11 infection. Disease symptoms were scored after 5 days according to Valent et al. (1991).

### Bioinformatics methods

We used Velvet version 1.1.04 (Zerbino and Birney, 2008) for *de novo* assembly of genome sequence. For ab initio gene prediction we used FgenesH (http://linux1.softberry.com/berry.phtml?topic=fgenesh). SignalP 3.0 (Bendtsen et al., 2004) and Phobius (Kall et al., 2004) were used for prediction of signal peptides and transmembrane domains. Alignments were visualized using the Artemis Comparison Tool (Carver et al., 2005). To generate Venn diagrams we used the Venn Diagram Generator (http://bioinformatics.psb.ugent.be/webtools/Venn/). PfamScan (Punta et al., 2012) was used to search for conserved domains in protein sequences.

### Linked ^1^H-NMR fingerprinting and direct infusion electrospray-mass spectrometry

Extracts from three replicate flasks of bran inoculum were prepared with equal 50 mL volumes of dH_2_0:acetic acid:methanol (80:20 vol/vol) and left to mix for 1 h at room temperature, prior to centrifugation at 12,000 × g, and subsequent filtration through 0.2 μm membranes (Millipore). Samples were snap frozen in liquid nitrogen and then lyophilized. Triplicate samples (15 mg) of freeze-dried *Trichoderma*-bran extract were processed for ^1^H-NMR-MS using modified protocols described by Ward et al. (2010). For ^1^H-NMR fingerprinting samples were extracted (10 min, 50°C) with 1 mL of deuterated sodium phosphate buffer (300 mM pH6) containing 0.05% w/v TSP-d4 (sodium salt of trimethylsilylpropionic acid). After centrifugation the supernatant was heated to 90°C (2 min). After cooling and centrifugation, the supernatant (600 μL) was transferred to an NMR tube for analysis. Samples for direct infusion electrospray mass spectrometry (DI-ESI-MS) were prepared exactly as described in Ward et al. (2010) and accompanying supplementary material using a mixture (20:80) methanol:water.

^1^H-NMR spectra were acquired under automation at a temperature of 300 K on an Avance spectrometer (Bruker Biospin) operating at 600.0528 MHz using a 5 mm SEI probe. ESI spectra were acquired, in both positive and negative ionization modes, under automation using Esquire 3000 (Bruker Daltonics) ion trap mass spectrometer.

^1^H-NMR spectra were automatically reduced to CSV files using AMIX (v3.0, Bruker Biospin) and DI-ESI-MS data were processed using Data Analysis v3.2 (Bruker Daltonics). Spectral processing for both ^1^H-NMR and DI-ESI-MS was carried out using routines described previously in Ward et al. (2010). Unsupervised multivariate analyses by PCA and PLS-DA were performed using SIMCA-P 11.0 (Umetrics, http://www.umetrics.com), using mean-centered scaling throughout the modeling. The signals resulting from the NMR internal standard (trimethylsilylpropionic acid; TSP-d_4_) were removed prior to importing the data set into SIMCA-P 11.0 for multivariate analysis.

## Results

### *Trichoderma hamatum* plant growth promotion

**Figure 1:**
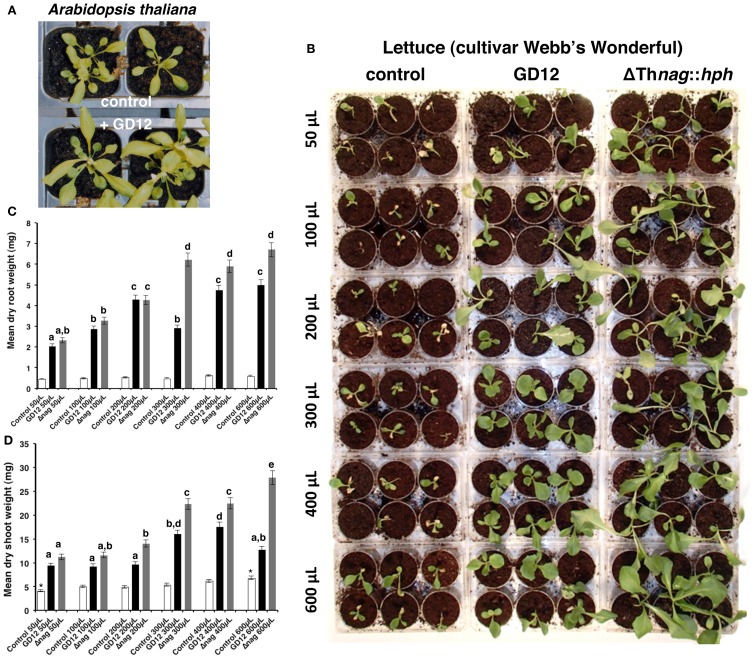
**Plant growth promotion by *Trichoderma hamatum* GD12. (A)** Amendment of peat compost with *T. hamatum* GD12 promotes growth of *Arabidopsis thaliana* accession Landsberg—erects. Photographed at 3 weeks. **(B)** Soluble, autoclaved bran extracts from GD12 or the *N*-acetyl-β-D-glucosaminidase knockout mutant (Δ*Thnag1::hph*) promote growth of lettuce (*Lactuca sativa* cultivar Webb's Wonderful) in sterile peat. Lettuce were supplemented with the indicated amount of sterilized bran exudate on alternate days. Control plants were watered with a corresponding aliquot of dH_2_O. After 21 days growth plants were harvested to determine root and shoot weights. Each microcosm diameter is 15 mm. The photograph, taken 21 days after sowing is representative of a microcosm replicate. Histogram showing dry weights of lettuce root **(C)** or shoot **(D)** biomass 21 days after growth in peat microcosms supplemented by application of sterilized bran extracts. Each plant was treated with the indicated amount of metabolite extract from either *T. hamatum* strain GD12 (black bars) or Δ*Thnag::hph* (Δnag; gray bars) on alternate days. Control plants (white bars) were watered with dH_2_O. Each bar represents the mean of 25 samples, each with 3 experimental replicates ± SE. Same letter denote no significant difference and ^*^denote significant difference at 95% confidence level (*t*-test).

*T. hamatum* promotes growth of lettuce (Ryder et al., 2012). Here we also demonstrate *T. hamatum* GD12 also promotes growth of *Arabidopsis thaliana* (Figure 1A) using acidic, nutrient-poor, organic peat soil microcosms (Thornton, 2005). In previous work, we hypothesized that the plant-growth-promotion (PGP) properties of GD12 might occur through the enzymatic release of nitrogen from chitin. Remarkably, however, rather than reduced or loss of PGP, disruption of the GD12 *N*-acetyl-b-D-glucosaminidase gene (Δ*Thnag1::hph* strain) dramatically enhanced the growth of lettuce seedlings, indicating that increased production of stimulatory compound(s) was due to *N*-acetyl-β-D-glucosaminidase deficiency (Ryder et al., 2012). Here we further demonstrate that this PGP activity was found to be present in water-soluble extracts derived from both bran grown GD12 and Δ*Thnag1::hph.* PGP activity, and that the PGP activity was heat stable, withstanding autoclaving to 121°C for 15 min (Figure 1B). Δ*Thnag1::hph* bran extracts promoted enhanced lettuce growth compared to GD12, which is consistent with the predicted hyper-secretion capability of Δ*Thnag1::hph*. These data were quantified by measurement of dry weights of treated material (Figures 1C,D). Application of either extract significantly increased PGP, even at the lowest addition (50 μL). The root dry weight of both GD12 and Δ*Thnag1::hph* treated lettuce showed significant increases to 200 μL aliquots application whereas Δ*Thnag1::hph* treatment root dry weight increased up to 300 μL aliquot applications (Figure 1C). Dry shoot weights followed a similar trend except PGP induced by GD12 extracts plateaued at 300 μL application and lettuce shoot dry weight continued to increase with 600 μL of extract (Figure 1D).

**Figure 2:**
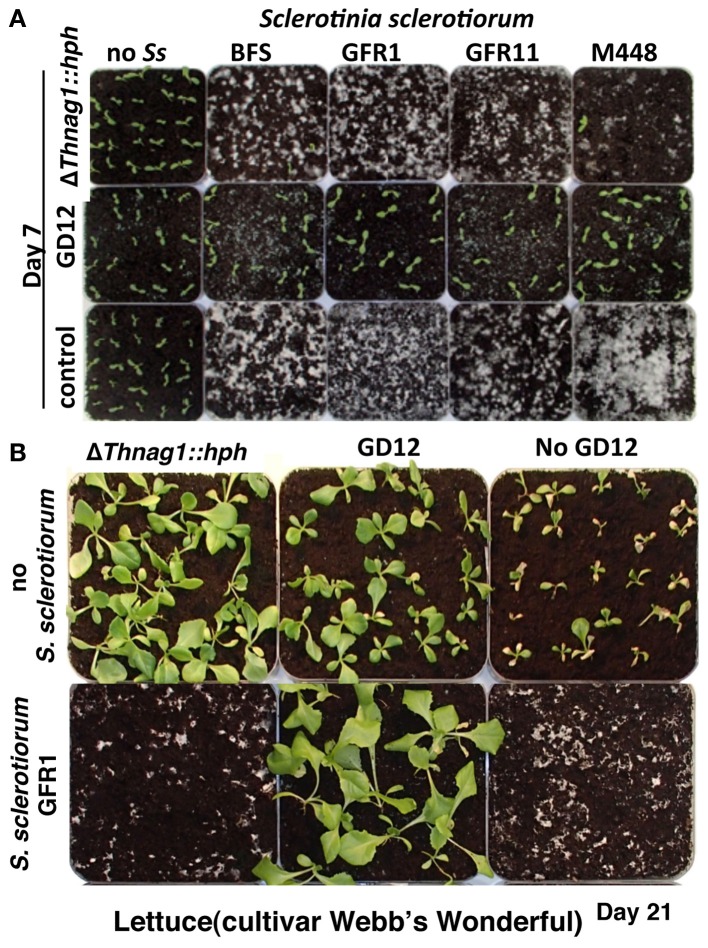
**Active biocontrol of the pre-emergence pathogen, *Sclerotinia sclerotiorum* by *T. hamatum*, GD12 results in additional plant growth promotion. (A)** *T. hamatum*, GD12 but not the *N*-acetyl-β-D-glucosaminidase mutant Δ*Thnag1::hph* is able to suppress *S. sclerotiorum* and allow germination of lettuce seedlings. Photograph taken 7 days post sowing. **(B)** Mycoparasitism of *Sclerotinia sclerotiorum* by *T. hamatum*, GD12 results in enhanced plant growth promotion, compared to amendment with GD12 alone.

It has been previously shown that in contrast to enhanced PGP, loss of *N*-acetyl-β-D-glucosaminidase activity drastically impaired GD12's competitive saprotrophic ability and biocontrol against a strain of the pre-emergent pathogen *S. sclerotiorum* (Ryder et al., 2012). We extended this study to show that GD12, but not Δ*Thnag1::hph*, effectively mycoparasitises four geographically distinct *Sclerotinia sclerotiorum* strains (Figure 2A). *T. hamatum* GD12 not only showed strong biocontrol of *S. sclerotiorum*, but strikingly PGP was also dramatically enhanced compared to GD12 amendment alone (Figure 2B). We interpret these data to suggest that cryptic metabolomic pathways, ordinarily silent in GD12 in axenic culture, are induced during antagonistic interactions in soil.

### *T. hamatum* induces resistance to rice blast

**Figure 3:**
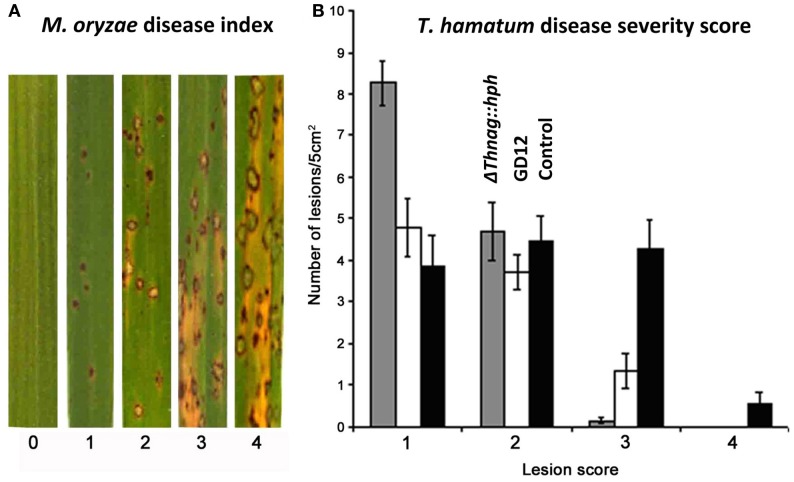
***T. hamatum* biocontrol and plant disease suppression. (A)** Leaf segments of rice (cultivar CO-39) showing rice blast symptoms. **(B)** Suppression of rice blast disease by *T. hamatum*. Growth of rice cultivar CO-39 in soil amended with *T. hamatum* GD12 (white bars) and the *N*-acetyl-β-D-glucosaminidase deficient mutant Δ*Thnag1::hph* (gray bars) reduced the size of the lesions caused by the rice blast *Magnaporthe oryzae*. Lesions were scored as previously documented (Valent et al., 1991) according to the following range. Type 1 (lesion 0.5 mm in length); type 2 (lesion ~1 mm in length); type 3 (lesions ~2 mm in length) and type 4 (lesions ~3–4 mm in length) lesions. Each bar represents the mean of 8 samples, each with 5 experimental replicates ± SE.

Production of plant-growth-promoting compounds and mycoparasitism by certain strains of *Trichoderma* are well-documented (Contreras-Cornejo et al., 2009; Vinale et al., 2009) although both traits are rarely found together. Some *Trichoderma* strains additionally possess the ability to activate ISR to a broad range of pathogens. To investigate the robustness of *T. hamatum* GD12 biocontrol properties we examined the ability of GD12 and the Δ*Thnag1::hph* mutant to confer resistance to the rice blast pathogen *Magnaporthae oryzae*. GD12 and, to a greater extent, Δ*Thnag1::hph* both reduced lesion formation compared to non-inoculated rice plants. Thus, although Δ*Thnag1::hph* has lost the ability to mycoparasitize *S. sclerotiorum* it has the capacity to elicit a strong induced systemic resistance response in rice to *M. oryzae* (Figure 3). Unlike the lettuce response, we did not see any clear increase in foliar growth, but observed an increase in root development following *Trichoderma* amendment.

### Draft genome sequence of *T. hamatum* GD12

We hypothesized that the genome sequence of *T. hamatum* GD12 might provide valuable insight into the genetic potential underlying the unique PGP, mycoparasitism and ISR inducing properties of this saprotrophic fungus. A GD12 genome sequence could facilitate secondary metabolite pathway predictions, mapping of mRNA-seq data to genomic clusters and capture unique genes and gene families not coded by other *Trichoderma* genomes.

We therefore assembled a draft genome sequence of GD12 from 12 million pairs of Illumina GA2 paired-end 73-bp reads using Velvet 1.1.04. This yielded 2770 scaffolds with a N_50_ length of 41.6 Kb. The total length of the assembly was 38.2 Mb. The whole genome shotgun data have been deposited at DDBJ/EMBL/GenBank under the accession ANCB00000000. Using FgenesH (trained on *Neurospora crassa*) we predicted 12391 protein-coding genes in GD12.

### Comparison with other *Trichoderma* genomes

The genome sequence of GD12 shares little similarity with previously sequenced *Trichoderma* genomes at the nucleotide sequence level. The three sequenced *Trichoderma* genomes analysed in detail, *T. atroviride* (~36.4 Mb), *T. virens* (~38.8 Mb) and *T. reesei* (~34 Mb) show remarkably conserved gene order (78–96%), with >50% of annotated genes having orthologues in the related ascomycetes *Neurospora crassa* and *Gibberella zeae.* (Kubicek et al., 2011). Strikingly, only 52% of the GD12 genome sequence aligned against that of *T. atroviride* and only 6% aligned against the more distantly related *T. reesei* (using the *dnadiff* tool from the Mummer package). To ensure this limited sequence similarity was not due to sample contamination, a geographically distinct *T. hamatum* isolate, strain 11, was sequenced. Strain 11 showed 98% sequence identify to GD12.

**Figure 4:**
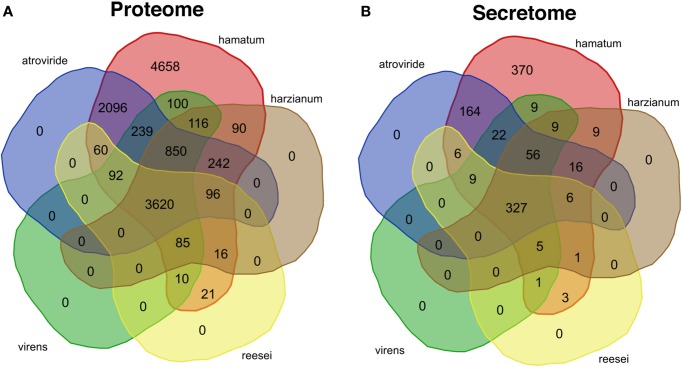
**Venn diagrams showing the conservation of the *Trichoderma hamatum* GD12 predicted proteome and secretome in previously sequenced *Trichoderma* species**. BLASTP was used to search for similar sequences to each of the 12,391 predicted GD12 proteins. We performed BLASTP searches against the previously published predicted proteomes of *T. atroviride*, *T. virens*, *T. reesei*, and *T. harzianum* as well as against the GD12 predicted proteome. A protein was counted as conserved in a species if there was a BLASTP hit with least 80% amino acid sequence identity covering at least 90% of the query sequence. The predicted proteins and a subset comprising the predicted secreted proteins were compared to other sequenced *Trichoderma* isolates. **(A)** *T. hamatum* GD12 shares a core proteome of 3620 predicted proteins, with *T. hamatum* with *T. atroviride*, *T. harzianum*, *T. reesei* and *T. virens* and has 4658 unique proteins. The GD12 proteome is most homologous to that of *T. atroviride*. **(B)** The 1,014 proteins predicted to encode secreted proteins based upon secretion signals (SignalP) and lack of a typical transmembrane domain (Phobius) were compared to similarly derived secretomes from *T. atroviride*, *T. harzianum*, *T. reesei* and *T. virens.* GD12 shares a core secretome of 327 proteins and has 370 predicted unique secreted proteins.

At the level of amino acid sequence, 62.4% of the GD12 predicted proteins (*i.e.* 7773 proteins; Figure 4A) had a close homologue in at least one of *T. atroviride*, *T. harzianum*, *T. reesei* or *T. virens* species compared (here, we define a close homologue as sharing at least 80% sequence identity over at least 90% of the length of the query sequence). Of the GD12 predicted proteins, only 5531 (59%) are highly conserved in *T. atroviride* (at least 80% amino acid sequence identity over at least 90% of the sequence length; Figure 4A). Thus, GD12 contains novel genomic regions with the potential to encode novel, agrochemically important gene products leading to unique bioactive metabolites that may contribute to GD12's PGP and biocontrol activities.

**Figure 5:**
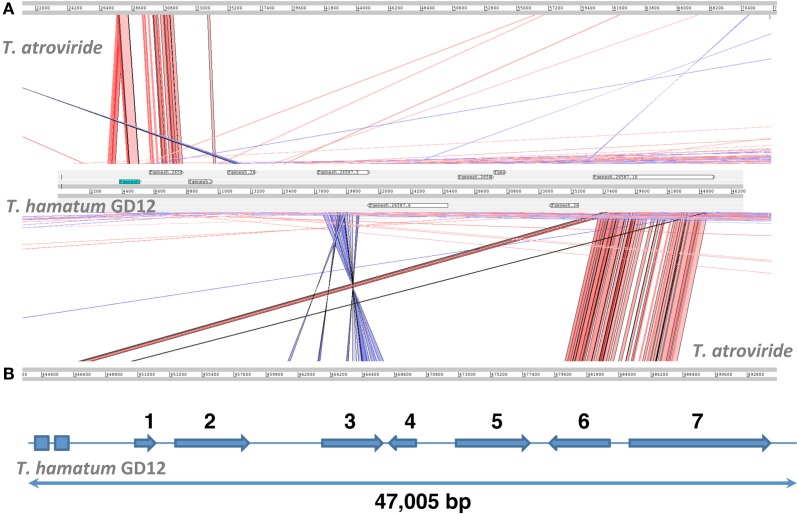
**A genomic region unique to *Trichoderma hamatum* GD12 with the capacity to encode novel secondary metabolites. (A)** This 47-kbp region (GenBank: KB232787) has no detectable nucleotide sequence similarity to previously sequenced *Trichoderma* genomes except for the two short regions indicated by rectangles, which share 85% and 78% nucleotide sequence identity with *T. atroviride* scaffold 19. **(B)** Arrows indicating predicted protein-coding genes, which are described in Table 1.

These *T. hamatum*-specific genomic regions likely hold the key to the unique biological interactions observed in this species. For example, we identified a 47-kbp *T. hamatum*-specific region described in Table 1 and illustrated in Figure 5, which appears to encode several enzymes (Genes 3, 5, 6, 7) and transporters (Genes 2, 4) that might contribute to novel secondary metabolism pathways. Predicted gene 3 encodes a protein containing an attachment site for phosphopantetheine, a prosthetic group that acts as a “swinging arm” for the attachment of activated fatty acid and amino-acid groups. It also contains a domain characteristic of AMP-binding enzymes. Gene 5 encodes a protein with an ATP-grasp domain, characteristic of enzymes that possess ATP-dependent carboxylate-amine ligase activity. Gene 6 encodes a putative aminotransferase while Gene 7 is predicted to encode a polyketide synthase, which provide important sources of naturally occurring small molecules such as antibiotics and other industrially important polyketides.

**Table 1:** **Genes encoded in a 47-kbp genomic region unique to *Trichoderma hamatum* GD12**.

**Gene**	**Start–end (orientation)**	**Best hit in SwissProt (amino acid sequence identity)**	**Best hit in NCBI Proteins**	**Pfam domains**
1	6,264–7,541 (+)	Q9PKX8.1 *Chlamydia muridarum* tyrosine-tRNA ligase (28%)	EGU81361.1 *Fusarium oxysporum* hypothetical protein (39%)	None
2	8,951–13,442 (+)	P11636.2 *Neurospora crassa* Quinate permease (32%)	EHK41798.1 *Trichoderma atroviride* hypothetical protein (60%)	Sugar (and other) transporter (PF00083)
3	17,805–21,557 (+)	Q4WYG2.2 *Aspergillus fumigatus* nonribosomal peptide synthetase 5 (31%)	XP_001262961.1 *Neosartorya fischeri* nonribosomal peptide synthase (54%)	AMP-binding enzyme (PF00501); Phosphopantetheine attachment site (PF00550)
4	21,747–23,653 (−)	Q864R9.1 *Macaca fascicularis* multidrug resistance-associated protein 1 (32%)	EHK16312.1 *Trichoderma virens* hypothetical protein (82%)	ABC transporter (PF00664); ABC transporter transmembrane region (PF00005)
5	26,142–30,603 (+)	Q6ZPS2.2 *Mus musculus* carnosine synthase 1 (26%)	XP_001262963.1 *Neosartorya fischeri* hypothetical protein (56%)	ATP-grasp domain (PF13535)
6	32,148–35,686 (−)	Q635G4.1 *Bacillus cereus* L-alanine–pimeloyl-CoA ligase (35%)	XP_003298955.1 *Pyrenophora teres f. teres* hypothetical protein (64%)	Aminotransferase class I and II (PF00155)
7	36,727–45,336 (+)	Q4WAZ9.2 *Aspergillus fumigatus* nonribosomal peptide synthetase 14 (38%)	ELA23575.1 *Colletotrichum gloeosporioides* polyketide synthase (49%)	Beta-ketoacyl synthase, N-terminal domain (PF00109);
				Beta-ketoacyl synthase, C-terminal domain (PF02801);
				Acyl transferase domain (PF00698);
				Alcohol dehydrogenase GroES-like domain (PF08240);
				Zinc-binding dehydrogenase (PF00107);
				KR domain (PF08659);
				Phosphopantetheine attachment site (PF00550)

This region (GenBank: KB232787) has no detectable nucleotide sequence similarity to previously sequenced Trichoderma genomes.

While the vast majority of the unique GD12 genes were of unknown function, there were some interesting candidate genes that might contribute to both PGP and biocontrol activities. These included enzymes with homology to benzene and benzoate oxidases and nicotinate (nicotinamidase) degradation; potential synthesis of the plant phytohormone zeatin (adenylate isopentenyltransferase) and the insect hormone biosynthetic hormone ecdysone oxidase. Most notable are the non-ribosomally synthesized cyclic lipopetide antibiotics such as surfactin and three of the five *Bacillus subtilis* fengycin synthetases that non-ribosomally synthesize fengycin, a lipopeptidic antibiotic (Wu et al., 2007). Thus, a rich reservoir of metabolic potential exists in the unique genomic regions of *T. hamatum.*

### The secretome

Given the dual plant growth promotion and biocontrol properties of *T. hamatum*, constituents of the “secretome” represent candidates in the molecular dialogue between soil pathogens and the plant rhizosphere. Of the 12391 hypothetical GD12 genes, 1014 (8.2%) were predicted to be encode secreted proteins based upon SignalP 3.0 (Bendtsen et al., 2004) and absence of a typical transmembrane domain as determined by Phobius (Kall et al., 2004) (Supplementary File S1). Of these, 370 were unique to *T. hamatum*, more than entire the “core” secretome shared by the 5 *Trichoderma* species (Figure 4B). Only 469 (55.5%) of *T. hamatum* secretome proteins are conserved in *T. atroviride* suggesting some divergence in the nature of the secreted bioactive proteins. The secretome of *T. atroviride* is, surprisingly, enriched for 26 proteins containing the fungal-specific Zn(2)Cys(6) transcription factor domain (Pfam: PF04082; http://genomebiology.com/2011/12/4/R40). The predicted secretome of GD12 is also similarly enriched, with 11 proteins containing this transcription-factor domain. One striking feature of the GD12 secretome is the enrichment for putative AMP-binding enzymes; 14 of the GD12 secreted proteins contain an AMP-binding domain (Pfam: PF00501; Supplementary File S2).

### Small secreted (cysteine-rich) proteins (SSCRPs)

Fungi manipulate the immune systems of their plant hosts via effector proteins, many of which are small secreted cysteine-rich proteins (Stergiopoulos and de Wit, 2009). Furthermore, it was recently shown that SSCRPs are upregulated in *Trichoderma* species during mycoparasitic interaction (Atanasova et al., 2013). We identified potential SSCRPs in the predicted secretomes of *T. hamatum* and *T. atroviride* as proteins whose length was 300 amino acids or fewer and which contained at least four cysteine residues, as defined in (Kubicek et al., 2011). There were 153 proteins in *T. hamatum* satisfying these criteria (Supplementary File S3), of which 83 had no close homologue in *T. harzianum*, *T. reesei*, *T. virens* or *T. atroviride* (i.e., no BLASTP hit with at least 80% sequence identity over at least 90% of the protein's length). For comparison, in *T. atroviride* there were 170 proteins satisfying these criteria, of which 106 had no close homologue in *T. harzianum*, *T. reesei*, *T. virens* or *T. hamatum*. Thus, there is a complement of ~60–70 SSCRPs that may constitute a “core” effector complement. The *T. hamatum* SSCRP's contained a diverse range of Pfam domains (Supplementary Files S4, S5) suggesting a complex array of biological activities associated with these SSCRPs.

### LysM motifs

The LysM motif binds different peptidoglycans in bacteria and chitin-like compounds in eukaryotes (Buist et al., 2008; de Jonge and Thomma, 2009). Recent studies have shown that fungal LysM motifs can bind and suppress chitin oligomers that would be recognized by plant pattern recognition receptors, preventing the activation of an innate immune response (de Jonge et al., 2010). Seven hypothetical GD12 proteins contain a LysM domain (Pfam: PF01476) although none of these are predicted to be secreted. This is similar to the numbers reported previously for *T. reesei* (6), *T. virens* (7) and *T. atroviride* (9).

### Metabolite profiling of the GD12 and ΔThnag1::hph mutant secreted metabolome

As a foundation to establish the underlying chemical differences that may collectively contribute to the PGP and biocontrol properties of *T. hamatum* we used two metabolite fingerprinting approaches, ^1^H-NMR and direct infusion electrospray ionization mass spectrometry (DI-ESI-MS). These methodologies sample a subset of overlapping, largely polar, chemistry which is ideally suited to analysis of the *T. hamatum* amended bran extracts showing PGP activity (Figure 2). Freeze dried, bran culture filtrates from uninoculated or GD12 and Δ*Thnag1::hph* amended cultures were initially extracted in 20% deuterated methanol in deuterium oxide solvent which is selective for polar metabolites but also has the advantage of being suitable for direct ^1^H-NMR analysis (Ward et al., 2003) and which we have successfully used in fingerprinting *Arabidopsis-Pseudomonas syringae* interactions (Ward et al., 2010). However, due to some shifts in peaks, presumably due to differing pHs of the samples, the freeze-dried bran culture filtrates were re-extracted using deuterated sodium phosphate buffer which aligned all peaks in the spectrum.

**Figure 6:**
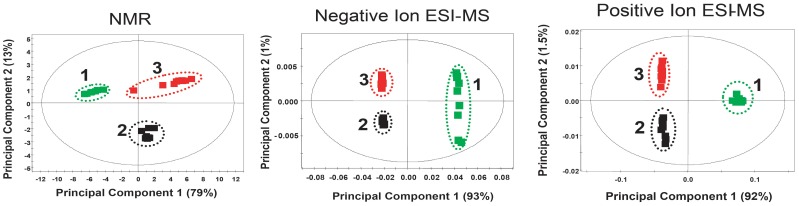
**Metabolite fingerprinting of bran extracts using NMR and DI-ESI-MS datasets**. Extracts from 5 day old *T. hamatum* bran inoculum were analysed by ^1^H-NMR and direct infusion electrospray ionization mass spectrometry. Principal components analysis (PCA) of full unfiltered data at 95% confidence intervals was used to evaluate differences in chemistry between the bran extracts. PCA data showed clear separation of GD12 (black) and Δ*Thnag1::hph* (red) from control bran extract green indicating both chemistries captured differences in the secreted metabolome. This unsupervised multivariate PCA analysis was performed using SIMCA-P 11.0, using mean-centered scaling.

Principal components analysis (PCA) of full unfiltered data at 95% confidence intervals was used to evaluate differences in chemistry between the bran extracts. Figure 6 show that both chemistries clearly showed statistically significant separation between the three treatments. Notably, the GD12 amended bran culture extract fingerprint is significantly different from Δ*Thnag1::hph* confirming differences in the secreted metabolome between the two that may contribute to the PGP differences illustrated in Figure 2. As part of a large scale metabolite profiling study of GD12 secreted metabolites we undertook unbiased metabolite profiling on GD12 bran extracts. Bran extracts prepared as above were first tested for plant growth promotion activity then three independent experiments, each containing 4 replicates were analysed by liquid chromatography mass spectrometry on a C18 column as described in Supplementary File S6. Spectra were extracted and features aligned from biological replicates using a density maximisation approach as previously described (Perera et al., 2012). This study confirmed a large number of potentially novel metabolites were secreted by GD12. Supplementary File S7 reports the top 30 ranked features identified in both positive and negative ionisation mode from GD12 bran extract showing at least a 5-fold enrichment in a minimum of 10 out of 12 samples analysed.

## Discussion and conclusions

Here we extend our initial characterization of *T. hamatum* strain GD12 (Ryder et al., 2012) to examine further beneficial agronomic traits of GD12. We show plant growth promotion in the model dicot, *Arabidopsis thaliana* and the ability to induce foliar biocontrol in rice, a model monocot. We further characterized the lettuce-*T. hamatum* interaction to show that bran extracts from GD12 and the *N*-acetyl-β-D-glucosamindase-deficient mutant Δ*Thnag1::hph* differentially promote growth in a concentration dependent manner. Both ^1^H-NMR and direct infusion electrospray ionization mass spectrometry fingerprinting show that these differences in growth are also correlated with differences in the small molecule secretome extracted from bran cultures. This finding was extended by unbiased comparative metabolite profiling of GD12 amended and un-amended bran extracts. We also expanded our recent finding of GD12 mycoparasitism of a strain of the pre-emergence soil pathogen *Sclerotinia sclerotiorum* to show mycoparasitism across a range of *S. sclerotiorum* strains. This interaction induces a further increase in plant growth promotion above that conferred by GD12 suggesting that cryptic metabolomic pathways, ordinarily silent in GD12 in axenic culture, are induced during antagonistic interactions in soil.

Fossil evidence predicts that the mycoparasitic lifestyle evolved more than 400 million years ago (Taylor and Berbee, 2006). We sequenced the *T. hamatum* genome and demonstrate that like *T. virens* and *T. atroviride*, *T. hamatum* encodes a vast arsenal of cell wall degrading enzymes such as chitinases, glycoside hydrolases, β −1,3-glucanases and *N*-acetyl-β-D-glucosaminidases that are presumably deployed to degrade the carbohydrate defenses of its biocontrol targets. It also encodes a wealth of proteases, polyketide synthases and non-peptide synthases consistent with its mycoparasitic lifestyle. Strikingly, however, approximately half of the *T. hamatum* proteome and its constituent secretome is unique to GD12 and *vice versa*. To ensure these data were not the result of unexpected contamination we sequenced the related *T. hamatum* strain 11 and verified ~98% identity.

Recent comparative genomic experiments have revealed that the *T. reesei* genome contraction has occurred, with consequent loss of mycoparasitic ability. At 38.8 Mbp, the *T. virens* genome is nearly 5 Mbp larger than *T. reesei* and 2.7 Mbp larger than *T. atroviride*. Unique to *T. virens* and *T. atroviride* are secondary metabolite gene clusters localized on non-syntenic islands that are likely to contribute to mycoparasitism. Notable in the larger *T. virens* genome, are a repertoire of non-ribosomal peptide synthetases (NRPS) expanded to 28, twice that present in other fungi (Martinez et al., 2008; Kubicek et al., 2011).

*T. hamatum* is phylogenetically most closely related to *T. atroviride* (Kubicek et al., 2011) yet the incomplete GD12 assembly at ~38.1 Mbp is nearly as large as the *T. virens* genome. Despite the close phylogenetic relationship, there were striking differences between *T. atroviride* and *T. hamatum* homology, with approximately 40% of the GD12 proteome being unique. The extent of these differences are interesting and most likely reflect the strong evolutionarily genomic potential and additional beneficial traits of plant growth promotion and induced systemic resistance encoded by GD12, as well as components required for niche differentiation. A simple analysis of GD12 unique regions identified a range of components implicated in secondary metabolism including evidence for production of non-ribosomally synthesized lipopetide antibiotics such as surfactin and the antibiotic fengycin (Wu et al., 2007).

*T. hamatum* encoded over 4658 unique proteins and shared a core proteome of 3620 predicted proteins with the four other *Trichoderma*'s (*T. atroviride, T. virens, T. reesei, T. harzianum*). An additional 2096 proteins were unique to GD12 and *T. atroviride* reflecting the closer evolutionarily relationship between these two species. Some of these genes may specify enzymes responsible for the breakdown of polymeric organic molecules into a form that can be absorbed, or in the secretion of fungal synthesized compounds that have roles in antibiosis or are signals molecules facilitating communication with mutualistic partners. How the unique component of the GD12 genome has been acquired and is deployed remains to be determined. Figure 5 highlights a 47-kbp *T. hamatum*-specific region that encodes several biosynthetic enzymes and transporters with potential to contribute to novel secondary chemistries, including two NRPS components. Moreover, like *T. atroviride* (Baker et al., 2012), the *T. hamatum* genome has a number gene clusters encoding polyketide synthases. PKSs play important roles in synthesis of secondary metabolites such as in the plant pathogen *Ustilago maydis* (Kamper et al., 2006) and a hybrid NRPS/PKS has recently been implicated in ISR in maize (Mukherjee et al., 2012).

We predicted 370 unique proteins in the secretome of *T. hamatum* and a core of 327 proteins shared across *T. atroviride, T. virens, T. reesei* and *T. harzianum.* GD12 and *T. atroviride* shared 164 unique putative secreted proteins, nearly 20 times as many as any of the other species (Figure 4). The distinct genomic potential is also reflected in the deployment of small secreted cysteine rich peptides which function as potential fungal effector proteins to suppress host immunity and modulate host signalling networks (Stergiopoulos and de Wit, 2009). Approximately 50% of the SSCRPs were shared between the two species.

A striking feature of the GD12 secretome was the enrichment for putative AMP-binding enzymes; 14 of the GD12 secreted proteins contain the AMP-binding domain (Pfam: PF00501; Supplementary File S1). Interestingly, many of these proteins are capable of acyl:adenyl ligase activities that can positively or negatively modulate bioactivity through the ligation of residues such as amino acids. Plant acyl:adenyl ligases include enzymes generating bioactive amide hormone conjugates such as JA-Ile and JA-Trp from JA and IAA-Trp from indole acetic acid (Staswick and Tiryaki, 2004; Staswick, 2009). Virulent phytopathogens such as *Pseudomonas syringae* synthesize IAA-lysine synthetase which can inactivate plant IAA to IAA-lysine (Romano et al., 1991). The AMP-binding domain containing *Cochliobolus carbonum* race 1 HC-toxin synthetase produces the HC-toxin cyclic tetrapeptide (Scott-Craig et al., 1992; Walton, 2006). Thus enrichment for putative secreted AMP-binding enzymes may highlight a possible role in plant-microbe communication in the rhizosphere.

Overall, it is an exciting and opportune time to exploit the remarkable genetic and chemical potential for beneficial for sustainable agriculture. Co-evolution with hosts has endowed *Trichoderma* spp. with a range of agronomically important traits. The genome of *T. hamatum* GD12 encodes the genetic potential to promote growth and induce ISR in a range of plants. The arsenal of genes in GD12 also enables it to effectively mycoparasitize *S. sclerotiorum*, a successful and persistent pathogen of agronomic crops. Strikingly, mycoparasitism of *S. sclerotiorum* results in additional PGP. Importantly, culture filtrates that promote plant growth (Figure 1B) are incapable of suppressing the pathogenic effects of *Sclerotinia* suggesting that, additional enhanced plant growth stimulation occurs during interactions with soil pathogens. We hypothesize that antagonism between GD12 and root pathogens in the plant rhizosphere leads to transcriptional activation of cryptic secondary metabolite pathways that are phenotypically silent in axenic culture. This is in agreement with recent reports of activation of silent gene clusters in *Aspergillus nidulans* following co-cultivation of the fungus with other microorganisms which has led to the identification of novel secondary metabolites (Schroeckh et al., 2009). However, it is also possible that mycoparasitism gives rise to new compounds from degrading tissue or releases pre-existing components with PGP activity. Thus, the genome sequence of GD12, and comparisons with other *Trichoderma* genomes will facilitate genetic dissection of these traits. Genome informed predictions will help to identify and experimentally validate novel secondary metabolism implicated in adaptation to specific ecological niches and promotion of beneficial traits.

### Conflict of interest statement

The authors declare that the research was conducted in the absence of any commercial or financial relationships that could be construed as a potential conflict of interest.

## Supplementary materials

Supplementary File S1 **GD12.secretome.no-TMs.faa.pfamscan.html**. Frequencies of Pfam domains in predicted secreted proteins encoded in the GD12 genome.

Supplementary File S2 **Supp2_AMP-binding_secreted.faa.txt**. AMP-binding domain proteins are over-represented in the secretome.

Supplementary File S3 **Supp3_GD12.secretome.no-TMs.SSCRPs.faa.txt**. Sequences of candidate small secreted cysteine-rich proteins (SSCRPs) encoded in the GD12 genome. Amino acid sequences are given in FastA format.

Supplementary File S4 **Supp4_GD12.secretome.no-TMs.SSCRPs.faa.pfamscan.txt**. Pfam domains in each predicted small secreted cysteine-rich proteins (SSCRPs) encoded in the GD12 genome.

Supplementary File S5 **Supp5_GD12.secretome.noTMs.SSCRPs.faa.pfamscan.html**. Frequencies of Pfam domains in predicted small secreted cysteine-rich proteins (SSCRPs) encoded in the GD12 genome.

Supplementary File S6 Method for unbiased profiling of GD12 and bran extracts by HPLC-MS.

Supplementary File S7 Table of top 30 putative metabolites identified in GD12 bran extracts showing at least a 5 fold change compared to unamended bran extract.
