# Supplementary material for: Investigating the beneficial traits of Trichoderma hamatum GD12 for sustainable agriculture—insights from genomics
Source: Front Plant Sci. 2013 Jul 30;4:258. doi: 10.3389/fpls.2013.00258 (PMC3726867; doi:10.3389/fpls.2013.00258)
Supplement: Supplementary File S1 — GD12.secretome.no-TMs.faa.pfamscan.html. Frequencies of Pfam domains in predicted secreted proteins encoded in the GD12 genome. [file DataSheet1.ZIP › Supplemental material/Supp1_PGP by extracts.pdf]

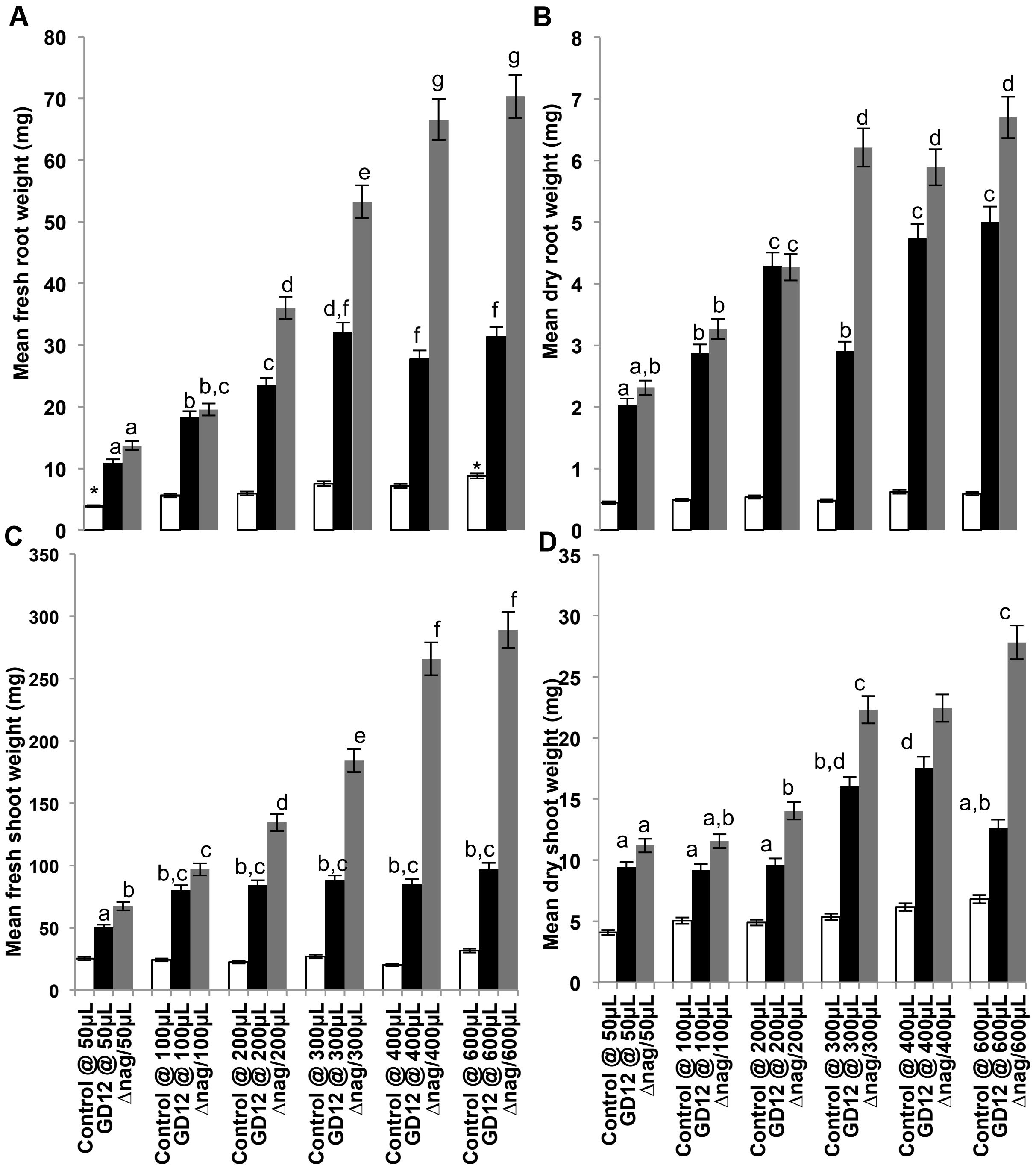

Supplemental Figure 1 Histogram showing fresh and dry weights of lettuce root/shoot biomass 21 days after growth in peat microcosms supplemented by application of sterilised bran extracts. Each plant was treated with the indicated amount of metabolite extract from either *T. hamatum* strain GD12 (black bars) or ∆*Thnag::hph* (∆*nag*; grey bars) on alternate days. Control plants (white bars) were watered with dH_20. Each bar represents the mean of 25 samples, each with 3 experimental replicates ± SE. Same letter denote no significant difference at 95% confidence level (t-test).
