# Supplementary material for: Investigating the beneficial traits of Trichoderma hamatum GD12 for sustainable agriculture—insights from genomics
Source: Front Plant Sci. 2013 Jul 30;4:258. doi: 10.3389/fpls.2013.00258 (PMC3726867; doi:10.3389/fpls.2013.00258)
Supplement: Supplementary File S1 — GD12.secretome.no-TMs.faa.pfamscan.html. Frequencies of Pfam domains in predicted secreted proteins encoded in the GD12 genome. [file DataSheet1.ZIP › Supplemental material/Supp2_GD12.secretome.no-TMs.faa.pfamscan.html]

|  |  |  |  |  |
| --- | --- | --- | --- | --- |
| 14 | PF00501 | AMP-binding | AMP-binding enzyme |  |
| 11 | PF04082 | Fungal\_trans | Fungal specific transcription factor domain |  |
| 8 | PF11951 | Fungal\_trans\_2 | Fungal specific transcription factor domain | This family of are likely to be transcription factors. This protein is found in fungi. Proteins in this family are typically between 454 to 826 amino acids in length. This protein is found associated with Pfam:PF00172. |
| 8 | PF00153 | Mito\_carr | Mitochondrial carrier protein |  |
| 6 | PF00107 | ADH\_zinc\_N | Zinc-binding dehydrogenase |  |
| 6 | PF00083 | Sugar\_tr | Sugar (and other) transporter |  |
| 5 | PF00550 | PP-binding | Phosphopantetheine attachment site | A 4'-phosphopantetheine prosthetic group is attached through a serine. This prosthetic group acts as a a 'swinging arm' for the attachment of activated fatty acid and amino-acid groups. This domain forms a four helix bundle. This family includes members not included in Prosite. The inclusion of these members is supported by sequence analysis and functional evidence. The related domain of Swiss:P19828 has the attachment serine replaced by an alanine. |
| 5 | PF00067 | p450 | Cytochrome P450 | Cytochrome P450s are haem-thiolate proteins [6] involved in the oxidative degradation of various compounds. They are particularly well known for their role in the degradation of environmental toxins and mutagens. They can be divided into 4 classes, according to the method by which electrons from NAD(P)H are delivered to the catalytic site. Sequence conservation is relatively low within the family - there are only 3 absolutely conserved residues - but their general topography and structural fold are highly conserved. The conserved core is composed of a coil termed the 'meander', a four-helix bundle, helices J and K, and two sets of beta-sheets. These constitute the haem-binding loop (with an absolutely conserved cysteine that serves as the 5th ligand for the haem iron), the proton-transfer groove and the absolutely conserved EXXR motif in helix K. While prokaryotic P450s are soluble proteins, most eukaryotic P450s are associated with microsomal membranes. their general enzymatic function is to catalyse regiospecific and stereospecific oxidation of non-activated hydrocarbons at physiological temperatures [6]. |
| 5 | PF07971 | Glyco\_hydro\_92 | Glycosyl hydrolase family 92 | Members of this family are alpha-1,2-mannosidases, enzymes which remove alpha-1,2-linked mannose residues from Man(9)(GlcNAc)(2) by hydrolysis. They are critical for the maturation of N-linked oligosaccharides and ER-associated degradation [1]. |
| 5 | PF00069 | Pkinase | Protein kinase domain |  |
| 5 | PF00005 | ABC\_tran | ABC transporter | ABC transporters for a large family of proteins responsible for translocation of a variety of compounds across biological membranes. ABC transporters are the largest family of proteins in many completely sequenced bacteria. ABC transporters are composed of two copies of this domain and two copies of a transmembrane domain Pfam:PF00664. These four domains may belong to a single polypeptide as in Swiss:P13569, or belong in different polypeptide chains. |
| 4 | PF01048 | PNP\_UDP\_1 | Phosphorylase superfamily | Members of this family include: purine nucleoside phosphorylase (PNP) Uridine phosphorylase (UdRPase) 5'-methylthioadenosine phosphorylase (MTA phosphorylase) |
| 4 | PF13561 | adh\_short\_C2 | Enoyl-(Acyl carrier protein) reductase |  |
| 4 | PF00271 | Helicase\_C | Helicase conserved C-terminal domain | The Prosite family is restricted to DEAD/H helicases, whereas this domain family is found in a wide variety of helicases and helicase related proteins. It may be that this is not an autonomously folding unit, but an integral part of the helicase. |
| 4 | PF00175 | NAD\_binding\_1 | Oxidoreductase NAD-binding domain | Xanthine dehydrogenases, that also bind FAD/NAD, have essentially no similarity. |
| 4 | PF00082 | Peptidase\_S8 | Subtilase family | Subtilases are a family of serine proteases. They appear to have independently and convergently evolved an Asp/Ser/His catalytic triad, like that found in the trypsin serine proteases (see Pfam:PF00089). Structure is an alpha/beta fold containing a 7-stranded parallel beta sheet, order 2314567. |
| 4 | PF00106 | adh\_short | short chain dehydrogenase | This family contains a wide variety of dehydrogenases. |
| 3 | PF00920 | ILVD\_EDD | Dehydratase family |  |
| 3 | PF00566 | RabGAP-TBC | Rab-GTPase-TBC domain | Identification of a TBC domain in GYP6\_YEAST and GYP7\_YEAST, which are GTPase activator proteins of yeast Ypt6 and Ypt7, implies that these domains are GTPase activator proteins of Rab-like small GTPases. |
| 3 | PF00258 | Flavodoxin\_1 | Flavodoxin |  |
| 3 | PF00441 | Acyl-CoA\_dh\_1 | Acyl-CoA dehydrogenase, C-terminal domain | C-terminal domain of Acyl-CoA dehydrogenase is an all-alpha, four helical up-and-down bundle. |
| 2 | PF08386 | Abhydrolase\_4 | TAP-like protein | This is a family of putative bacterial peptidases and hydrolases that bear similarity to a tripeptidyl aminopeptidase isolated from Streptomyces lividans (Swiss:Q54410). A member of this family (Swiss:Q6E3K7) is thought to be involved in the C-terminal processing of propionicin F, a bacteriocidin characterised from Propionibacterium freudenreichii [1]. |
| 2 | PF04303 | PrpF | PrpF protein | PrpF is a protein found in the 2-methylcitrate pathway. It is structurally similar to DAP epimerase and proline racemase. This protein is likely to acts to isomerise trans-aconitate to cis-aconitate [1]. |
| 2 | PF02775 | TPP\_enzyme\_C | Thiamine pyrophosphate enzyme, C-terminal TPP binding domain |  |
| 2 | PF00072 | Response\_reg | Response regulator receiver domain | This domain receives the signal from the sensor partner in bacterial two-component systems. It is usually found N-terminal to a DNA binding effector domain. |
| 2 | PF04389 | Peptidase\_M28 | Peptidase family M28 |  |
| 2 | PF00734 | CBM\_1 | Fungal cellulose binding domain |  |
| 2 | PF01798 | Nop | Putative snoRNA binding domain | This family consists of various Pre RNA processing ribonucleoproteins. The function of the aligned region is unknown however it may be a common RNA or snoRNA or Nop1p binding domain. Nop5p (Nop58p) Swiss:Q12499 from yeast is the protein component of a ribonucleoprotein protein required for pre-18s rRNA processing and is suggested to function with Nop1p in a snoRNA complex [1]. Nop56p Swiss:O00567 and Nop5p interact with Nop1p and are required for ribosome biogenesis [2]. Prp31p Swiss:p49704 is required for pre-mRNA splicing in S. cerevisiae [3]. |
| 2 | PF00814 | Peptidase\_M22 | Glycoprotease family | The Peptidase M22 proteins are part of the HSP70-actin superfamily ([1]). The region represented here is an insert into the fold and is not found in the rest of the family (beyond the Peptidase M22 family). Included in this family are the Rhizobial NodU proteins and the HypF regulator. This region also contains the histidine dyad believed to coordinate the metal ion and hence provide catalytic activity. Interestingly the histidines are not well conserved, and there is a lack of experimental evidence to support peptidase activity as a general property of this family. There also appear to be instances of this domain outside of the HSP70-actin superfamily (e.g. Swiss:Q9ZM49). |
| 2 | PF02615 | Ldh\_2 | Malate/L-lactate dehydrogenase | This family consists of bacterial and archaeal Malate/L-lactate dehydrogenase. L-lactate dehydrogenase, EC:1.1.1.27, catalyses the reaction (S)-lactate + NAD(+) <=> pyruvate + NADH. Malate dehydrogenase, EC:1.1.1.37 and EC:1.1.1.82, catalyses the reactions: (S)-malate + NAD(+) <=> oxaloacetate + NADH, and (S)-malate + NADP(+) <=> oxaloacetate + NADPH respectively. |
| 2 | PF06766 | Hydrophobin\_2 | Fungal hydrophobin | This is a family of fungal hydrophobins that seems to be restricted to ascomycetes. These are small, moderately hydrophobic extracellular proteins that have eight cysteine residues arranged in a strictly conserved motif. Hydrophobins are generally found on the outer surface of conidia and of the hyphal wall, and may be involved in mediating contact and communication between the fungus and its environment [1]. Note that some family members contain multiple copies. |
| 2 | PF00664 | ABC\_membrane | ABC transporter transmembrane region | This family represents a unit of six transmembrane helices. Many members of the ABC transporter family (Pfam:PF00005) have two such regions. |
| 2 | PF01494 | FAD\_binding\_3 | FAD binding domain | This domain is involved in FAD binding in a number of enzymes. |
| 2 | PF00654 | Voltage\_CLC | Voltage gated chloride channel | This family of ion channels contains 10 or 12 transmembrane helices. Each protein forms a single pore. It has been shown that some members of this family form homodimers. In terms of primary structure, they are unrelated to known cation channels or other types of anion channels. Three ClC subfamilies are found in animals. ClC-1 (Swiss:P35523) is involved in setting and restoring the resting membrane potential of skeletal muscle, while other channels play important parts in solute concentration mechanisms in the kidney [3]. These proteins contain two Pfam:PF00571 domains. |
| 2 | PF03452 | Anp1 | Anp1 | The members of this family (Anp1, Van1 and Mnn9) are membrane proteins required for proper Golgi function. These proteins co-localise within the cis Golgi, and that they are physically associated in two distinct complexes[1]. |
| 2 | PF00704 | Glyco\_hydro\_18 | Glycosyl hydrolases family 18 |  |
| 2 | PF02463 | SMC\_N | RecF/RecN/SMC N terminal domain | This domain is found at the N terminus of SMC proteins. The SMC (structural maintenance of chromosomes) superfamily proteins have ATP-binding domains at the N- and C-termini, and two extended coiled-coil domains separated by a hinge in the middle. The eukaryotic SMC proteins form two kind of heterodimers: the SMC1/SMC3 and the SMC2/SMC4 types. These heterodimers constitute an essential part of higher order complexes, which are involved in chromatin and DNA dynamics [1]. This family also includes the RecF and RecN proteins that are involved in DNA metabolism and recombination. |
| 2 | PF02734 | Dak2 | DAK2 domain | This domain is the predicted phosphatase domain of the dihydroxyacetone kinase family. |
| 2 | PF03953 | Tubulin\_C | Tubulin C-terminal domain | This family includes the tubulin alpha, beta and gamma chains. Members of this family are involved in polymer formation. Tubulins are GTPases. FtsZ can polymerise into tubes, sheets, and rings in vitro and is ubiquitous in eubacteria and archaea. Tubulin is the major component of microtubules. (The FtsZ GTPases have been split into their won family). |
| 2 | PF01055 | Glyco\_hydro\_31 | Glycosyl hydrolases family 31 | Glycosyl hydrolases are key enzymes of carbohydrate metabolism. Family 31 comprises of enzymes that are, or similar to, alpha- galactosidases. |
| 2 | PF01008 | IF-2B | Initiation factor 2 subunit family | This family includes initiation factor 2B alpha, beta and delta subunits from eukaryotes, initiation factor 2B subunits 1 and 2 from archaebacteria and some proteins of unknown function from prokaryotes. Initiation factor 2 binds to Met-tRNA, GTP and the small ribosomal subunit. Members of this family have also been characterised as 5-methylthioribose- 1-phosphate isomerases, an enzyme of the methionine salvage pathway. The crystal structure of Ypr118w, a non-essential, low-copy number gene product from Saccharomyces cerevisiae, reveals a dimeric protein with two domains and a putative active site cleft [2]. |
| 2 | PF00155 | Aminotran\_1\_2 | Aminotransferase class I and II |  |
| 2 | PF00400 | WD40 | WD domain, G-beta repeat |  |
| 2 | PF14295 | PAN\_4 | PAN domain |  |
| 2 | PF12417 | DUF3669 | Zinc finger protein | This domain family is found in eukaryotes, and is typically between 64 and 80 amino acids in length. |
| 2 | PF00378 | ECH | Enoyl-CoA hydratase/isomerase family | This family contains a diverse set of enzymes including: Enoyl-CoA hydratase (Swiss:Q13011). Napthoate synthase (Swiss:P27290). Carnitate racemase (Swiss:P31551). 3-hydoxybutyryl-CoA dehydratase (Swiss:P52046). Dodecanoyl-CoA delta-isomerase (Swiss:P42126). |
| 2 | PF09770 | PAT1 | Topoisomerase II-associated protein PAT1 | Members of this family are necessary for accurate chromosome transmission during cell division [1]. |
| 2 | PF10294 | Methyltransf\_16 | Putative methyltransferase |  |
| 2 | PF03313 | SDH\_alpha | Serine dehydratase alpha chain | L-serine dehydratase (EC:4.2.1.13) is a found as a heterodimer of alpha and beta chain or as a fusion of the two chains in a single protein. This enzyme catalyses the deamination of serine to form pyruvate. This enzyme is part of the gluconeogenesis pathway. |
| 2 | PF06398 | Pex24p | Integral peroxisomal membrane peroxin | Peroxisomes play diverse roles in the cell, compartmentalising many activities related to lipid metabolism and functioning in the decomposition of toxic hydrogen peroxide. Sequence similarity was identified between two hypothetical proteins and the peroxin integral membrane protein Pex24p [1]. |
| 2 | PF00667 | FAD\_binding\_1 | FAD binding domain | This domain is found in sulfite reductase, NADPH cytochrome P450 reductase, Nitric oxide synthase and methionine synthase reductase. |
| 2 | PF05277 | DUF726 | Protein of unknown function (DUF726) | This family consists of several uncharacterised eukaryotic proteins. |
| 2 | PF00022 | Actin | Actin |  |
| 2 | PF00171 | Aldedh | Aldehyde dehydrogenase family | This family of dehydrogenases act on aldehyde substrates. Members use NADP as a cofactor. The family includes the following members: The prototypical members are the aldehyde dehydrogenases Swiss:P00352 EC:1.2.1.3. Succinate-semialdehyde dehydrogenase Swiss:P25526 EC:1.2.1.16. Lactaldehyde dehydrogenase Swiss:P25553 EC:1.2.1.22. Benzaldehyde dehydrogenase Swiss:P43503 EC:1.2.1.28. Methylmalonate-semialdehyde dehydrogenase Swiss:Q02252 EC:1.2.1.27. Glyceraldehyde-3-phosphate dehydrogenase Swiss:P81406 EC:1.2.1.9. Delta-1-pyrroline-5-carboxylate dehydrogenase Swiss:P30038 EC: 1.5.1.12. Acetaldehyde dehydrogenase Swiss:P17547 EC:1.2.1.10. Glutamate-5-semialdehyde dehydrogenase Swiss:P07004 EC:1.2.1.41. This family also includes omega crystallin Swiss:P30842 an eye lens protein from squid and octopus that has little aldehyde dehydrogenase activity. |
| 2 | PF12796 | Ank\_2 | Ankyrin repeats (3 copies) |  |
| 2 | PF00076 | RRM\_1 | RNA recognition motif. (a.k.a. RRM, RBD, or RNP domain) | The RRM motif is probably diagnostic of an RNA binding protein. RRMs are found in a variety of RNA binding proteins, including various hnRNP proteins, proteins implicated in regulation of alternative splicing, and protein components of snRNPs. The motif also appears in a few single stranded DNA binding proteins. The RRM structure consists of four strands and two helices arranged in an alpha/beta sandwich, with a third helix present during RNA binding in some cases The C-terminal beta strand (4th strand) and final helix are hard to align and have been omitted in the SEED alignment The LA proteins (Swiss:P05455) have an N terminal rrm which is included in the seed. There is a second region towards the C terminus that has some features characteristic of a rrm but does not appear to have the important structural core of a rrm. The LA proteins (Swiss:P05455) are one of the main autoantigens in Systemic lupus erythematosus (SLE), an autoimmune disease. |
| 1 | PF00571 | CBS | CBS domain | CBS domains are small intracellular modules that pair together to form a stable globular domain [2]. This family represents a single CBS domain. Pairs of these domains have been termed a Bateman domain [6]. CBS domains have been shown to bind ligands with an adenosyl group such as AMP, ATP and S-AdoMet [5]. CBS domains are found attached to a wide range of other protein domains suggesting that CBS domains may play a regulatory role making proteins sensitive to adenosyl carrying ligands. The region containing the CBS domains in Cystathionine-beta synthase is involved in regulation by S-AdoMet [4]. CBS domain pairs from AMPK bind AMP or ATP [5]. The CBS domains from IMPDH and the chloride channel CLC2 bind ATP [5]. |
| 1 | PF04051 | TRAPP | Transport protein particle (TRAPP) component | TRAPP plays a key role in the targeting and/or fusion of ER-to-Golgi transport vesicles with their acceptor compartment. TRAPP is a large multimeric protein that contains at least 10 subunits. This family contains many TRAPP family proteins. The Bet3 subunit is one of the better characterised TRAPP proteins and has a dimeric structure [2] with hydrophobic channels. The channel entrances are located on a putative membrane-interacting surface that is distinctively flat, wide and decorated with positively charged residues. Bet3 is proposed to localise TRAPP to the Golgi [2]. |
| 1 | PF04117 | Mpv17\_PMP22 | Mpv17 / PMP22 family | The 22-kDa peroxisomal membrane protein (PMP22) is a major component of peroxisomal membranes. PMP22 seems to be involved in pore forming activity and may contribute to the unspecific permeability of the organelle membrane. PMP22 is synthesised on free cytosolic ribosomes and then directed to the peroxisome membrane by specific targeting information [1]. Mpv17 is a closely related peroxisomal protein. In mouse, the Mpv17 protein is involved in the development of early-onset glomerulosclerosis [2]. More recently a homolog of Mpv17 in S. cerevisiae has been been found to be an integral membrane protein of the inner mitochondrial membrane where it has been proposed to have a role in ethanol metabolism and tolerance during heat-shock [3]. Defects in MPV17 is associated with mitochondrial DNA depletion syndrome (MDDS) and Navajo neurohepatopathy (NNH) [4][5]. MDDS is a clinically heterogeneous group of disorders characterised by a reduction in mitochondrial DNA (mtDNA) copy number. Primary mtDNA depletion is inherited as an autosomal recessive trait and may affect single organs, typically muscle or liver, or multiple tissues. Individuals with the hepatocerebral form of mitochondrial DNA depletion syndrome have early progressive liver failure and neurologic abnormalities, hypoglycemia, and increased lactate in body fluids. NNH is an autosomal recessive disease that is prevalent among Navajo children in the South Western states of America. The major clinical features are hepatopathy, peripheral neuropathy, corneal anesthesia and scarring, acral mutilation, cerebral leukoencephalopathy, failure to thrive, and recurrent metabolic acidosis with intercurrent infections. Infantile, childhood, and classic forms of NNH have been described. Mitochondrial DNA depletion was detected in the livers of patients, suggesting a primary defect in mtDNA maintenance [5]. |
| 1 | PF10233 | Cg6151-P | Uncharacterized conserved protein CG6151-P | This is a family of small, less than 200 residue long, proteins which are named as CG6151-P proteins that are conserved from fungi to humans. The function is unknown. The fungal members have a characteristic ICP sequence motif. Some members are annotated as putative clathrin-coated vesicle protein but this could not be defined. |
| 1 | PF11022 | DUF2611 | Protein of unknown function (DUF2611) | This family is conserved in the Dikarya of Fungi. The function is not known. |
| 1 | PF01058 | Oxidored\_q6 | NADH ubiquinone oxidoreductase, 20 Kd subunit |  |
| 1 | PF12739 | TRAPPC-Trs85 | ER-Golgi trafficking TRAPP I complex 85 kDa subunit | This family is one of the subunits of the TRAPP Golgi trafficking complex. TRAPP subunits are found in two different sized complexes, TRAPP I and TRAPP II, and this Trs85 is in the smaller complex. TRAPP I, but Not TRAPP II, functions in ER-Golgi transport [1]. Trs85p was reported to function in the cytosol-to-vacuole targeting pathway, suggesting a role for this subunit in autophagy as well as in secretion [2]. The overall architecture of TRAPP I shows the other components to be Bet3p (TRAPPC3), Bet5p (TRAPPC1), Trs20p (TRAPPC2) , Trs23p (TRAPPC4), Trs31p (TRAPPC5), Trs33p (TRAPPC6a and b) and Trs85p. |
| 1 | PF04616 | Glyco\_hydro\_43 | Glycosyl hydrolases family 43 | The glycosyl hydrolase family 43 contains members that are arabinanase. Rabinanases hydrolyses the alpha-1,5-linked L-arabinofuranoside backbone of plant cell wall arabinans. The structure of arabinanase Arb43A from Cellvibrio japonicus reveals a five-bladed beta-propeller fold. A long V-shaped groove, partially enclosed at one end, forms a single extended substrate-binding surface across the face of the propeller [1]. |
| 1 | PF03031 | NIF | NLI interacting factor-like phosphatase | This family contains a number of NLI interacting factor isoforms (eg. Swiss:Q9PTJ8) and also an N-terminal regions of RNA polymerase II CTC phosphatase (Swiss:Q9Y5BO) and FCP1 serine phosphatase (Swiss:Q9PT70). This region has been identified as the minimal phosphatase domain [1]. |
| 1 | PF04055 | Radical\_SAM | Radical SAM superfamily | Radical SAM proteins catalyse diverse reactions, including unusual methylations, isomerisation, sulphur insertion, ring formation, anaerobic oxidation and protein radical formation. |
| 1 | PF01095 | Pectinesterase | Pectinesterase |  |
| 1 | PF10510 | PIG-S | Phosphatidylinositol-glycan biosynthesis class S protein | PIG-S is one of several key, core, components of the glycosylphosphatidylinositol (GPI) trans-amidase complex that mediates GPI anchoring in the endoplasmic reticulum. Anchoring occurs when a protein's C-terminal GPI attachment signal peptide is replaced with a pre-assembled GPI [1]. Mammalian GPITransamidase consists of at least five components: Gaa1, Gpi8, PIG-S, PIG-T, and PIG-U, all five of which are required for function. It is possible that Gaa1, Gpi8, PIG-S, and PIG-T form a tightly associated core that is only weakly associated with PIG-U. The exact function of PIG-S is unclear [2]. |
| 1 | PF00383 | dCMP\_cyt\_deam\_1 | Cytidine and deoxycytidylate deaminase zinc-binding region |  |
| 1 | PF12621 | DUF3779 | Phosphate metabolism protein | This domain family is found in eukaryotes, and is approximately 100 amino acids in length. The family is found in association with Pfam:PF02714. There are two completely conserved residues (W and D) that may be functionally important. This family is likely to be involved in phosphate metabolism however there is little accompanying literature to confirm this. |
| 1 | PF00433 | Pkinase\_C | Protein kinase C terminal domain |  |
| 1 | PF13639 | zf-RING\_2 | Ring finger domain |  |
| 1 | PF00291 | PALP | Pyridoxal-phosphate dependent enzyme | Members of this family are all pyridoxal-phosphate dependent enzymes. This family includes: serine dehydratase EC:4.2.1.13 P20132, threonine dehydratase EC:4.2.1.16 Swiss:P04968, tryptophan synthase beta chain EC:4.2.1.20 Swiss:P00932, threonine synthase EC:4.2.99.2 Swiss:P04990, cysteine synthase EC:4.2.99.8 P11096, cystathionine beta-synthase EC:4.2.1.22 Swiss:P35520, 1-aminocyclopropane-1-carboxylate deaminase EC:4.1.99.4 Swiss:P76316. |
| 1 | PF00462 | Glutaredoxin | Glutaredoxin |  |
| 1 | PF00025 | Arf | ADP-ribosylation factor family | Pfam combines a number of different Prosite families together |
| 1 | PF03849 | Tfb2 | Transcription factor Tfb2 |  |
| 1 | PF10075 | PCI\_Csn8 | COP9 signalosome, subunit CSN8 | This PCI\_Csn8 domain is conserved from plants to humans. It is a signature protein motif found in components of CSN (COP9 signalosome). It functions as a structural scaffold for subunit-subunit interactions within the complex and is a key regulator of photomorphogenic development [1]. |
| 1 | PF07859 | Abhydrolase\_3 | alpha/beta hydrolase fold | This catalytic domain is found in a very wide range of enzymes. |
| 1 | PF02666 | PS\_Dcarbxylase | Phosphatidylserine decarboxylase | This is a family of phosphatidylserine decarboxylases, EC:4.1.1.65. These enzymes catalyse the reaction: Phosphatidyl-L-serine <=> phosphatidylethanolamine + CO2. Phosphatidylserine decarboxylase plays a central role in the biosynthesis of aminophospholipids by converting phosphatidylserine to phosphatidylethanolamine [2]. |
| 1 | PF00542 | Ribosomal\_L12 | Ribosomal protein L7/L12 C-terminal domain |  |
| 1 | PF05368 | NmrA | NmrA-like family | NmrA is a negative transcriptional regulator involved in the post-translational modification of the transcription factor AreA. NmrA is part of a system controlling nitrogen metabolite repression in fungi [1]. This family only contains a few sequences as iteration results in significant matches to other Rossmann fold families. |
| 1 | PF00689 | Cation\_ATPase\_C | Cation transporting ATPase, C-terminus | Members of this families are involved in Na+/K+, H+/K+, Ca++ and Mg++ transport. This family represents 5 transmembrane helices. |
| 1 | PF13893 | RRM\_5 | RNA recognition motif. (a.k.a. RRM, RBD, or RNP domain) | The RRM motif is probably diagnostic of an RNA binding protein. RRMs are found in a variety of RNA binding proteins, including various hnRNP proteins, proteins implicated in regulation of alternative splicing, and protein components of snRNPs. The motif also appears in a few single stranded DNA binding proteins. |
| 1 | PF01507 | PAPS\_reduct | Phosphoadenosine phosphosulfate reductase family | This domain is found in phosphoadenosine phosphosulfate (PAPS) reductase enzymes or PAPS sulfotransferase. PAPS reductase is part of the adenine nucleotide alpha hydrolases superfamily also including N type ATP PPases and ATP sulphurylases [1]. The enzyme uses thioredoxin as an electron donor for the reduction of PAPS to phospho-adenosine-phosphate (PAP) [1,2]. It is also found in NodP nodulation protein P from Rhizobium which has ATP sulfurylase activity (sulfate adenylate transferase) [3]. |
| 1 | PF02260 | FATC | FATC domain | The FATC domain is named after FRAP, ATM, TRRAP C-terminal [1]. The solution structure of the FATC domain suggests it plays a role in redox-dependent structural and cellular stability [2]. |
| 1 | PF10176 | DUF2370 | Protein of unknown function (DUF2370) | This family is conserved from fungi to humans. The human member is annotated as a Golgi-associated protein-Nedd4 WW domain-binding protein but this could not be confirmed. |
| 1 | PF03630 | Fumble | Fumble | Fumble is required for cell division in Drosophila. Mutants lacking fumble exhibit abnormalities in bipolar spindle organisation, chromosome segregation, and contractile ring formation. Analyses have demonstrated that encodes three protein isoforms, all of which contain a domain with high similarity to the pantothenate kinases of A. nidulans and mouse[1]. A role of fumble in membrane synthesis has been proposed[1]. |
| 1 | PF13023 | HD\_3 | HD domain | HD domains are metal dependent phosphohydrolases. |
| 1 | PF13242 | Hydrolase\_like | HAD-hyrolase-like |  |
| 1 | PF00723 | Glyco\_hydro\_15 | Glycosyl hydrolases family 15 | In higher organisms this family is represented by phosphorylase kinase subunits. |
| 1 | PF07217 | Het-C | Heterokaryon incompatibility protein Het-C | In filamentous fungi, het loci (for heterokaryon incompatibility) are believed to regulate self/nonself-recognition during vegetative growth. As filamentous fungi grow, hyphal fusion occurs within an individual colony to form a network. Hyphal fusion can occur also between different individuals to form a heterokaryon, in which genetically distinct nuclei occupy a common cytoplasm. However, heterokaryotic cells are viable only if the individuals involved have identical alleles at all het loci [1]. |
| 1 | PF03169 | OPT | OPT oligopeptide transporter protein | The OPT family of oligopeptide transporters is distinct from the ABC Pfam:PF00005 and PTR Pfam:PF00854 transporter families. OPT transporters were first recognised in fungi (Candida albicans and Schizosaccharomyces pombe), but this alignment also includes orthologues from Arabidopsis thaliana. OPT transporters are thought to have 12-14 transmembrane domains and contain the following motif: SPYxEVRxxVxxxDDP [1]. |
| 1 | PF02045 | CBFB\_NFYA | CCAAT-binding transcription factor (CBF-B/NF-YA) subunit B |  |
| 1 | PF04148 | Erv26 | Transmembrane adaptor Erv26 | Erv26 is an integral membrane protein that is packed into COPII vesicles and cycles between the ER and Golgi compartments. It directs pro-alkaline phosphatase into endoplasmic reticulum-derived COPII transport vesicles [1]. |
| 1 | PF13405 | EF\_hand\_4 | EF-hand domain |  |
| 1 | PF03663 | Glyco\_hydro\_76 | Glycosyl hydrolase family 76 | Family of alpha-1,6-mannanases. |
| 1 | PF02518 | HATPase\_c | Histidine kinase-, DNA gyrase B-, and HSP90-like ATPase | This family represents the structurally related ATPase domains of histidine kinase, DNA gyrase B and HSP90. |
| 1 | PF01470 | Peptidase\_C15 | Pyroglutamyl peptidase |  |
| 1 | PF03595 | C4dic\_mal\_tran | C4-dicarboxylate transporter/malic acid transport protein | This family of transporters have ten alpha helical transmembrane segments [1]. The structure of a bacterial homologue of SLAC1 shows it to have a trimeric arrangement. The pore is composed of five helices with a conserved phe residue involved in gating. One homologue, Mae1 from the yeast Schizosaccharomyces pombe, functions as a malate uptake transporter; another, Ssu1 from Saccharomyces cerevisiae and other fungi including Aspergillus fumigatus, is characterized as a sulphite efflux pump; and TehA from Escherichia coli is identified as a tellurite resistance protein by virtue of its association in the tehA/tehB operon. Many homologues are incorrectly annotated as tellurite resistance/dicarboxylate transporter (TDT) proteins. |
| 1 | PF00149 | Metallophos | Calcineurin-like phosphoesterase | This family includes a diverse range of phosphoesterases [1], including protein phosphoserine phosphatases, nucleotidases, sphingomyelin phosphodiesterases and 2'-3' cAMP phosphodiesterases as well as nucleases such as bacterial SbcD Swiss:P13457 or yeast MRE11 Swiss:P32829. The most conserved regions in this superfamily centre around the metal chelating residues. |
| 1 | PF00565 | SNase | Staphylococcal nuclease homologue | Present in all three domains of cellular life. Four copies in the transcriptional coactivator p100: these, however, appear to lack the active site residues of Staphylococcal nuclease. Positions 14 (Asp-21), 34 (Arg-35), 39 (Asp-40), 42 (Glu-43) and 110 (Arg-87) [SNase numbering in parentheses] are thought to be involved in substrate-binding and catalysis. |
| 1 | PF00018 | SH3\_1 | SH3 domain | SH3 (Src homology 3) domains are often indicative of a protein involved in signal transduction related to cytoskeletal organisation. First described in the Src cytoplasmic tyrosine kinase Swiss:P12931. The structure is a partly opened beta barrel. |
| 1 | PF02544 | Steroid\_dh | 3-oxo-5-alpha-steroid 4-dehydrogenase | This family consists of 3-oxo-5-alpha-steroid 4-dehydrogenases, EC:1.3.99.5 Also known as Steroid 5-alpha-reductase, the reaction catalysed by this enzyme is: 3-oxo-5-alpha-steroid + acceptor <=> 3-oxo-delta(4)-steroid + reduced acceptor. The Steroid 5-alpha-reductase enzyme is responsible for the formation of dihydrotestosterone, this hormone promotes the differentiation of male external genitalia and the prostate during fetal development [2]. In humans mutations in this enzyme can cause a form of male pseudohermaphorditism in which the external genitalia and prostate fail to develop normally [2]. A related enzyme is also found in plants is Swiss:Q38944 (DET2) a steroid reductase from Arabidopsis. Mutations in this enzyme cause defects in light-regulated development [1]. |
| 1 | PF14420 | Clr5 | Clr5 domain | This domain is found at the N-terminus of the Clr5 protein which has been shown to be involved in silencing in fission yeast. This domain has been found to often be associated with proteins that contain ankyrin repeats and large regions of disordered sequence [1]. |
| 1 | PF14225 | MOR2-PAG1\_C | Cell morphogenesis C-terminal | This family is the conserved C-terminal region of proteins that are involved in cell morphogenesis. |
| 1 | PF02815 | MIR | MIR domain | The MIR (protein mannosyltransferase, IP3R and RyR) domain is a domain that may have a ligand transferase function [1]. |
| 1 | PF04258 | Peptidase\_A22B | Signal peptide peptidase | The members of this family are membrane proteins. In some proteins this region is found associated with Pfam:PF02225. This family corresponds with Merops subfamily A22B, the type example of which is signal peptide peptidase. There is a sequence-similarity relationship with Pfam:PF01080. |
| 1 | PF00319 | SRF-TF | SRF-type transcription factor (DNA-binding and dimerisation domain) |  |
| 1 | PF08566 | Pam17 | Mitochondrial import protein Pam17 | The presequence translocase-associated motor (PAM) drives the completion of preprotein translocation into the mitochondrial matrix. The Pam17 subunit is required for formation of a stable complex between cochaperones Pam16 and Pam18 and promotes the association of Pam16-Pam18 with the presequence translocase [1]. Mitochondria lacking Pam17 are selectively impaired in the import of matrix proteins [1]. |
| 1 | PF03070 | TENA\_THI-4 | TENA/THI-4/PQQC family | Members of this family are found in all the three major phyla of life: archaebacteria, eubacteria, and eukaryotes. In Bacillus subtilis, TENA is one of a number of proteins that enhance the expression of extracellular enzymes, such as alkaline protease, neutral protease and levansucrase [1]. The THI-4 protein, which is involved in thiamine biosynthesis, is also a member of this family. The C-terminal part of these proteins consistently show significant sequence similarity to TENA proteins. This similarity was first noted with the Neurospora crassa THI-4 [2]. This family includes bacterial coenzyme PQQ synthesis protein C or PQQC proteins. Pyrroloquinoline quinone (PQQ) is the prosthetic group of several bacterial enzymes,including methanol dehydrogenase of methylotrophs and the glucose dehydrogenase of a number of bacteria [3]. PQQC has been found to be required in the synthesis of PQQ but its function is unclear. The exact molecular function of members of this family is uncertain. |
| 1 | PF03747 | ADP\_ribosyl\_GH | ADP-ribosylglycohydrolase | This family includes enzymes that ADP-ribosylations, for example ADP-ribosylarginine hydrolase EC:3.2.2.19 cleaves ADP-ribose-L-arginine [1]. The family also includes dinitrogenase reductase activating glycohydrolase [2]. Most surprisingly the family also includes jellyfish crystallins [2], these proteins appear to have lost the presumed active site residues. |
| 1 | PF12697 | Abhydrolase\_6 | Alpha/beta hydrolase family | This family contains alpha/beta hydrolase enzymes of diverse specificity. |
| 1 | PF00211 | Guanylate\_cyc | Adenylate and Guanylate cyclase catalytic domain |  |
| 1 | PF00075 | RNase\_H | RNase H | RNase H digests the RNA strand of an RNA/DNA hybrid. Important enzyme in retroviral replication cycle, and often found as a domain associated with reverse transcriptases. Structure is a mixed alpha+beta fold with three a/b/a layers. |
| 1 | PF04950 | DUF663 | Protein of unknown function (DUF663) | This family contains several uncharacterised eukaryotic proteins. |
| 1 | PF00326 | Peptidase\_S9 | Prolyl oligopeptidase family |  |
| 1 | PF07748 | Glyco\_hydro\_38C | Glycosyl hydrolases family 38 C-terminal domain | Glycosyl hydrolases are key enzymes of carbohydrate metabolism. |
| 1 | PF11779 | DUF3317 | Protein of unknown function (DUF3317) | This is a short family of proteins conserved from fungi and plants to human. One each of the human and mouse members is annotated as being androgen down-regulated protein expressed in mouse prostate, with a potential signal transduction function, and all appear to be membrane proteins. |
| 1 | PF11707 | Npa1 | Ribosome 60S biogenesis N-terminal | Npa1p is required for ribosome biogenesis and operates in the same functional environment as Rsa3p and Dbp6p during early maturation of 60S ribosomal subunits [1]. The protein partners of Npa1p include eight putative helicases as well as the novel Npa2p factor. Npa1p can also associate with a subset of H/ACA and C/D small nucleolar RNPs (snoRNPs) involved in the chemical modification of residues in the vicinity of the peptidyl transferase centre [2]. The protein has also been referred to as Urb1, and this domain at the N-terminal is one of several conserved regions along the length. |
| 1 | PF00137 | ATP-synt\_C | ATP synthase subunit C |  |
| 1 | PF01931 | NTPase\_I-T | Protein of unknown function DUF84 | The function of this prokaryotic protein family is unknown. |
| 1 | PF13350 | Y\_phosphatase3 | Tyrosine phosphatase family | This family is closely related to the Pfam:PF00102 and Pfam:PF00782 families. |
| 1 | PF06472 | ABC\_membrane\_2 | ABC transporter transmembrane region 2 | This domain covers the transmembrane of a small family of ABC transporters and shares sequence similarity with Pfam:PF00664. Mutations in this domain in Swiss:P28288 are believed responsible for Zellweger Syndrome-2 [1]; mutations in Swiss:P33897 are responsible for recessive X-linked adrenoleukodystrophy [2]. A Saccharomyces cerevisiae homolog is involved in the import of long-chain fatty acids [3]. |
| 1 | PF02574 | S-methyl\_trans | Homocysteine S-methyltransferase | This is a family of related homocysteine S-methyltransferases enzymes: 5-methyltetrahydrofolate--homocysteine S-methyltransferases also known EC:2.1.1.13, [2]; Betaine--homocysteine S-methyltransferase (vitamin B12 dependent), EC:2.1.1.5, [3]; and Homocysteine S-methyltransferase, EC:2.1.1.10, [1]. |
| 1 | PF00009 | GTP\_EFTU | Elongation factor Tu GTP binding domain | This domain contains a P-loop motif, also found in several other families such as Pfam:PF00071, Pfam:PF00025 and Pfam:PF00063. Elongation factor Tu consists of three structural domains, this plus two C-terminal beta barrel domains. |
| 1 | PF00614 | PLDc | Phospholipase D Active site motif | Phosphatidylcholine-hydrolysing phospholipase D (PLD) isoforms are activated by ADP-ribosylation factors (ARFs). PLD produces phosphatidic acid from phosphatidylcholine, which may be essential for the formation of certain types of transport vesicles or may be constitutive vesicular transport to signal transduction pathways. PC-hydrolysing PLD is a homologue of cardiolipin synthase, phosphatidylserine synthase, bacterial PLDs, and viral proteins. Each of these appears to possess a domain duplication which is apparent by the presence of two motifs containing well-conserved histidine, lysine, and/or asparagine residues which may contribute to the active site. aspartic acid. An E. coli endonuclease (nuc) and similar proteins appear to be PLD homologues but possess only one of these motifs. The profile contained here represents only the putative active site regions, since an accurate multiple alignment of the repeat units has not been achieved. |
| 1 | PF09384 | UTP15\_C | UTP15 C terminal | U3 snoRNA is ubiquitous in eukaryotes and is required for nucleolar processing of pre-18S ribosomal RNA [1]. It is a component of the ribosomal small subunit (SSU) processome. UTP15 is needed for optimal pre-ribosomal RNA transcription by RNA polymerase I, together with a subset of U3 proteins required for transcription (t-UTPs) [2]. This entry represents the C terminal of UTP15, and is found adjacent to WD40 repeats (Pfam:PF00400). |
| 1 | PF14138 | COX16 | Cytochrome c oxidase assembly protein COX16 | This family represents homologues of COX16 [1] which has been shown to be involved in assembly of cytochrome oxidase [2]. Protein in this family are typically between 106 and 134 amino acids in length. |
| 1 | PF04116 | FA\_hydroxylase | Fatty acid hydroxylase superfamily | This superfamily includes fatty acid and carotene hydroxylases and sterol desaturases. Beta-carotene hydroxylase is involved in zeaxanthin synthesis by hydroxylating beta-carotene, but the enzyme may be involved in other pathways [1]. This family includes C-5 sterol desaturase and C-4 sterol methyl oxidase. Members of this family are involved in cholesterol biosynthesis and biosynthesis a plant cuticular wax. These enzymes contain two copies of a HXHH motif. Members of this family are integral membrane proteins. |
| 1 | PF06985 | HET | Heterokaryon incompatibility protein (HET) | This family represents a conserved region approximately 150 residues long within various heterokaryon incompatibility proteins that seem to be restricted to ascomycete fungi. Genetic differences in specific het genes prevent a viable heterokaryotic fungal cell from being formed by the fusion of filaments from two different wild-type strains [1]. Many family members also contain the Pfam:PF00400 repeat and the Pfam:PF05729 domain. |
| 1 | PF00487 | FA\_desaturase | Fatty acid desaturase |  |
| 1 | PF01485 | IBR | IBR domain | The IBR (In Between Ring fingers) domain is often found to occur between pairs of ring fingers (Pfam:PF00097). This domain has also been called the C6HC domain and DRIL (for double RING finger linked) domain [2]. Proteins that contain two Ring fingers and an IBR domain (these proteins are also termed RBR family proteins) are thought to exist in all eukaryotic organisms. RBR family members play roles in protein quality control and can indirectly regulate transcription [3]. Evidence suggests that RBR proteins are often parts of cullin-containing ubiquitin ligase complexes. The ubiquitin ligase Parkin is an RBR family protein whose mutations are involved in forms of familial Parkinson's disease [3][4]. |
| 1 | PF01399 | PCI | PCI domain | This domain has also been called the PINT motif (Proteasome, Int-6, Nip-1 and TRIP-15) [1]. |
| 1 | PF03060 | NMO | Nitronate monooxygenase | Nitronate monooxygenase (NMO), formerly referred to as 2-nitropropane dioxygenase (NPD) (EC:1.13.11.32), is an FMN-dependent enzyme that uses molecular oxygen to oxidize (anionic) alkyl nitronates and, in the case of the enzyme from Neurospora crassa, (neutral) nitroalkanes to the corresponding carbonyl compounds and nitrite. Previously classified as 2-nitropropane dioxygenase [1,2,3], but it is now recognized that this was the result of the slow ionization of nitroalkanes to their nitronate (anionic) forms [4]. The enzymes from the fungus Neurospora crassa and the yeast Williopsis saturnus var. mrakii (formerly classified as Hansenula mrakii) contain non-covalently bound FMN as the cofactor. Active towards linear alkyl nitronates of lengths between 2 and 6 carbon atoms and, with lower activity, towards propyl-2-nitronate. The enzyme from N. crassa can also utilize neutral nitroalkanes, but with lower activity. One atom of oxygen is incorporated into the carbonyl group of the aldehyde product. The reaction appears to involve the formation of an enzyme-bound nitronate radical and an a-peroxynitroethane species, which then decomposes, either in the active site of the enzyme or after release, to acetaldehyde and nitrite. |
| 1 | PF02770 | Acyl-CoA\_dh\_M | Acyl-CoA dehydrogenase, middle domain | Central domain of Acyl-CoA dehydrogenase has a beta-barrel fold. |
| 1 | PF04488 | Gly\_transf\_sug | Glycosyltransferase sugar-binding region containing DXD motif | The DXD motif is a short conserved motif found in many families of glycosyltransferases, which add a range of different sugars to other sugars, phosphates and proteins. DXD-containing glycosyltransferases all use nucleoside diphosphate sugars as donors and require divalent cations, usually manganese. The DXD motif is expected to play a carbohydrate binding role in sugar-nucleoside diphosphate and manganese dependent glycosyltransferases [1]. |
| 1 | PF03666 | NPR3 | Nitrogen Permease regulator of amino acid transport activity 3 | This family, also known in yeasts as Rmd11, complexes with NPR2, Pfam:PF06218. This complex heterodimer is responsible for inactivating TORC1. an evolutionarily conserved protein complex that controls cell size via nutritional input signals, specifically, in response to amino acid starvation. |
| 1 | PF01464 | SLT | Transglycosylase SLT domain | This family is distantly related to Pfam:PF00062. Members are found in phages, type II, type III and type IV secretion systems (reviewed in [4]). |
| 1 | PF01145 | Band\_7 | SPFH domain / Band 7 family | This family has been called SPFH [1], Band 7 or PHB domain. Recent phylogenetic analysis has shown this domain to be a slipin or Stomatin-like integral membrane domain conserved from protozoa to mammals. |
| 1 | PF00293 | NUDIX | NUDIX domain |  |
| 1 | PF14374 | Ribos\_L4\_asso\_C | 60S ribosomal protein L4 C-terminal domain | This family is found at the very C-terminal of 60 ribosomal L4 proteins. |
| 1 | PF03144 | GTP\_EFTU\_D2 | Elongation factor Tu domain 2 | Elongation factor Tu consists of three structural domains, this is the second domain. This domain adopts a beta barrel structure. This the second domain is involved in binding to charged tRNA [1]. This domain is also found in other proteins such as elongation factor G and translation initiation factor IF-2. This domain is structurally related to Pfam:PF03143, and in fact has weak sequence matches to this domain. |
| 1 | PF00125 | Histone | Core histone H2A/H2B/H3/H4 |  |
| 1 | PF10255 | Paf67 | RNA polymerase I-associated factor PAF67 | RNA polymerase I is a multisubunit enzyme and its transcription competence is dependent on the presence of PAF67 [1]. This family of proteins is conserved from worms to humans. |
| 1 | PF07247 | AATase | Alcohol acetyltransferase | This family contains a number of alcohol acetyltransferase (EC:2.3.1.84) enzymes approximately 500 residues long found in both bacteria and metazoa. These catalyse the esterification of isoamyl alcohol by acetyl coenzyme A [1]. |
| 1 | PF11635 | Med16 | Mediator complex subunit 16 | Mediator is a large complex of up to 33 proteins that is conserved from plants through fungi to humans - the number and representation of individual subunits varying with species [1-2]. It is arranged into four different sections, a core, a head, a tail and a kinase-activity part, and the number of subunits within each of these is what varies with species. Overall, Mediator regulates the transcriptional activity of RNA polymerase II but it would appear that each of the four different sections has a slightly different function. Med16 is one of the subunits of the Tail portion of the Mediator complex and is required for lipopolysaccharide gene-expression [4]. Several members including the human protein, Swiss:Q9Y2X0, have one or more WD40 domains on them, Pfam:PF00400. |
| 1 | PF00264 | Tyrosinase | Common central domain of tyrosinase | This family also contains polyphenol oxidases and some hemocyanins. Binds two copper ions via two sets of three histidines. This family is related to Pfam:PF00372. |
| 1 | PF01300 | Sua5\_yciO\_yrdC | Telomere recombination | This domain has been shown to bind preferentially to dsRNA [1]. The domain is found in SUA5 Swiss:P32579 as well as HypF and YrdC Swiss:P45748. It has also been shown to be required for telomere recombniation in yeast. |
| 1 | PF02854 | MIF4G | MIF4G domain | MIF4G is named after Middle domain of eukaryotic initiation factor 4G (eIF4G). Also occurs in NMD2p and CBP80. The domain is rich in alpha-helices and may contain multiple alpha-helical repeats. In eIF4G, this domain binds eIF4A, eIF3, RNA and DNA [1]. |
| 1 | PF06824 | DUF1237 | Protein of unknown function (DUF1237) | This family contains a number of hypothetical proteins of about 450 residues in length. Their function is unknown, and most are bacterial. However, structurally this family is part of the 6 hairpin glycosidase superfamily, suggesting a glycosyl hydrolase function. |
| 1 | PF13460 | NAD\_binding\_10 | NADH(P)-binding |  |
| 1 | PF02801 | Ketoacyl-synt\_C | Beta-ketoacyl synthase, C-terminal domain | The structure of beta-ketoacyl synthase is similar to that of the thiolase family (Pfam:PF00108) and also chalcone synthase. The active site of beta-ketoacyl synthase is located between the N and C-terminal domains. |
| 1 | PF01370 | Epimerase | NAD dependent epimerase/dehydratase family | This family of proteins utilise NAD as a cofactor. The proteins in this family use nucleotide-sugar substrates for a variety of chemical reactions. |
| 1 | PF00343 | Phosphorylase | Carbohydrate phosphorylase | The members of this family catalyse the formation of glucose 1-phosphate from one of the following polyglucoses; glycogen, starch, glucan or maltodextrin. |
| 1 | PF02102 | Peptidase\_M35 | Deuterolysin metalloprotease (M35) family |  |
| 1 | PF00012 | HSP70 | Hsp70 protein | Hsp70 chaperones help to fold many proteins. Hsp70 assisted folding involves repeated cycles of substrate binding and release. Hsp70 activity is ATP dependent. Hsp70 proteins are made up of two regions: the amino terminus is the ATPase domain and the carboxyl terminus is the substrate binding region. |
| 1 | PF03715 | Noc2 | Noc2p family | At least one member, Noc2p from yeast, is required for a late step in 60S subunit export from the nucleus [2]. It has also been shown to co-precipitate with Nug1p, a nuclear GTPase also required for ribosome nucleus export [1]. This family was formerly known as UPF0120. |
| 1 | PF00512 | HisKA | His Kinase A (phospho-acceptor) domain | Dimerisation and phospho-acceptor domain of histidine kinases. |
| 1 | PF01851 | PC\_rep | Proteasome/cyclosome repeat |  |
| 1 | PF05426 | Alginate\_lyase | Alginate lyase | This family contains several bacterial alginate lyase proteins. Alginate is a family of 1-4-linked copolymers of beta -D-mannuronic acid (M) and alpha -L-guluronic acid (G). It is produced by brown algae and by some bacteria belonging to the genera Azotobacter and Pseudomonas. Alginate lyases catalyse the depolymerisation of alginates by beta -elimination, generating a molecule containing 4-deoxy-L-erythro-hex-4-enepyranosyluronate at the nonreducing end [1]. This family adopts an all alpha fold [2]. |
| 1 | PF00122 | E1-E2\_ATPase | E1-E2 ATPase |  |
| 1 | PF01532 | Glyco\_hydro\_47 | Glycosyl hydrolase family 47 | Members of this family are alpha-mannosidases that catalyse the hydrolysis of the terminal 1,2-linked alpha-D-mannose residues in the oligo-mannose oligosaccharide Man(9)(GlcNAc)(2). |
| 1 | PF02585 | PIG-L | GlcNAc-PI de-N-acetylase | Members of this family are related to PIG-L an N-acetylglucosaminylphosphatidylinositol de-N-acetylase (EC:3.5.1.89) that catalyses the second step in GPI biosynthesis [1]. |
| 1 | PF12719 | Cnd3 | Nuclear condensing complex subunits, C-term domain | The Cnd1-3 proteins are the three non-SMC (structural maintenance of chromosomes) proteins that go to make up the mitotic condensation complex along with the two SMC protein families, XCAP-C and XCAP-E, (or in the case of fission yeast, Cut3 and Cut14). The five-member complex seems to be conserved from yeasts to vertebrates. This domain is the C-terminal, cysteine-rich domain of Cnd3. The complex shuttles between the nucleus, during mitosis, and the cytoplasm during the rest of the cycle. Thus this family is made up of the C-termini of XCAP-Gs, Ycg1 and Ycs5 members. |
| 1 | PF04828 | GFA | Glutathione-dependent formaldehyde-activating enzyme |  |
| 1 | PF00766 | ETF\_alpha | Electron transfer flavoprotein FAD-binding domain | This domain found at the C-terminus of electron transfer flavoprotein alpha chain and binds to FAD [1]. The fold consists of a five-stranded parallel beta sheet as the core of the domain, flanked by alternating helices. A small part of this domain is donated by the beta chain [1]. |
| 1 | PF03142 | Chitin\_synth\_2 | Chitin synthase | Members of this family are fungal chitin synthase EC:2.4.1.16 enzymes. They catalyse chitin synthesis as follows: UDP-N-acetyl-D-glucosamine + {(1,4)-(N-acetyl-beta-D-glucosaminyl)}(N) <=> UDP + {(1,4)-(N-acetyl-beta-D-glucosaminyl)}(N+1). |
| 1 | PF10785 | NADH-u\_ox-rdase | NADH-ubiquinone oxidoreductase complex I, 21 kDa subunit | This family is the N-terminal domain of NADH-ubiquinone oxidoreductase 21 kDa subunits from fungi, lower metazoa and plants. |
| 1 | PF01885 | PTS\_2-RNA | RNA 2'-phosphotransferase, Tpt1 / KptA family | Tpt1 catalyses the last step of tRNA splicing in yeast. It transfers the splice junction 2'-phosphate from ligated tRNA to NAD, to produce ADP-ribose 1"-2"-cyclic phosphate. This is presumed to be followed by a transesterification step to release the RNA. The first step of this reaction is similar to that catalysed by some bacterial toxins. E. coli KptA and mouse Tpt1 are likely to use the same reaction mechanism [1]. |
| 1 | PF06966 | DUF1295 | Protein of unknown function (DUF1295) | This family contains a number of bacterial and eukaryotic proteins of unknown function that are approximately 300 residues long. |
| 1 | PF08659 | KR | KR domain | This enzymatic domain is part of bacterial polyketide synthases and catalyses the first step in the reductive modification of the beta-carbonyl centres in the growing polyketide chain. It uses NADPH to reduce the keto group to a hydroxy group [1]. |
| 1 | PF07745 | Glyco\_hydro\_53 | Glycosyl hydrolase family 53 | This domain belongs to family 53 of the glycosyl hydrolase classification [1]. These enzymes are enzymes are endo-1,4- beta-galactanases (EC:3.2.1.89). The structure of this domain is known [2] and has a TIM barrel fold. |
| 1 | PF02110 | HK | Hydroxyethylthiazole kinase family |  |
| 1 | PF02887 | PK\_C | Pyruvate kinase, alpha/beta domain | As well as being found in pyruvate kinase this family is found as an isolated domain in some bacterial proteins. |
| 1 | PF03435 | Saccharop\_dh | Saccharopine dehydrogenase | This family comprised of three structural domains that can not be separated in the linear sequence. In some organisms this enzyme is found as a bifunctional polypeptide with lysine ketoglutarate reductase. The saccharopine dehydrogenase can also function as a saccharopine reductase. |
| 1 | PF09785 | Prp31\_C | Prp31 C terminal domain | This is the C terminal domain of the pre-mRNA processing factor Prp31. Prp31 is required for U4/U6.U5 tri-snRNP formation [2]. In humans this protein has been linked to autosomal dominant retinitis pigmentosa [2][3]. |
| 1 | PF00581 | Rhodanese | Rhodanese-like domain | Rhodanese has an internal duplication. This Pfam represents a single copy of this duplicated domain. The domain is found as a single copy in other proteins, including phosphatases and ubiquitin C-terminal hydrolases. |
| 1 | PF03481 | SUA5 | Putative GTP-binding controlling metal-binding | Structural investigation of this domain suggests that it might be a GTP-binding region that regulates metal binding and involves hydrolysis of ATP to AMP. It is found to the C-terminus of Pfam:PF01300. |
| 1 | PF04928 | PAP\_central | Poly(A) polymerase central domain | The central domain of Poly(A) polymerase shares structural similarity with the allosteric activity domain of ribonucleotide reductase R1, which comprises a four-helix bundle and a three-stranded mixed beta- sheet. Even though the two enzymes bind ATP, the ATP-recognition motifs are different. |
| 1 | PF04003 | Utp12 | Dip2/Utp12 Family | This domain is found at the C-terminus of proteins containing WD40 repeats. These proteins are part of the U3 ribonucleoprotein the yeast protein is called Utp12 or DIP2 Swiss:Q12220 [1]. |
| 1 | PF12853 | NADH\_u\_ox\_C | C-terminal of NADH-ubiquinone oxidoreductase 21 kDa subunit | This family is the C-terminal domain of NADH-ubiquinone oxidoreductase 21 kDa subunits from fungi. |
| 1 | PF09118 | DUF1929 | Domain of unknown function (DUF1929) | Members of this family adopt a secondary structure consisting of a bundle of seven, mostly antiparallel, beta-strands surrounding a hydrophobic core. The 7 strands are arranged in 2 sheets, in a Greek-key topology. Their precise function, has not, as yet, been defined, though they are mostly found in sugar-utilising enzymes, such as galactose oxidase [1]. |
| 1 | PF01425 | Amidase | Amidase |  |
| 1 | PF05922 | Inhibitor\_I9 | Peptidase inhibitor I9 | This family includes the proteinase B inhibitor from Saccharomyces cerevisiae and the activation peptides from peptidases of the subtilisin family. The subtilisin propeptides are known to function as molecular chaperones, assisting in the folding of the mature peptidase [1], but have also been shown to act as 'temporary inhibitors' [2]. |
| 1 | PF00285 | Citrate\_synt | Citrate synthase |  |
| 1 | PF13410 | GST\_C\_2 | Glutathione S-transferase, C-terminal domain | This domain is closely related to Pfam:PF00043. |
| 1 | PF00573 | Ribosomal\_L4 | Ribosomal protein L4/L1 family | This family includes Ribosomal L4/L1 from eukaryotes and archaebacteria and L4 from eubacteria. L4 from yeast has been shown to bind rRNA [1]. |
| 1 | PF05405 | Mt\_ATP-synt\_B | Mitochondrial ATP synthase B chain precursor (ATP-synt\_B) | The Fo sector of the ATP synthase is a membrane bound complex which mediates proton transport. It is composed of nine different polypeptide subunits (a, b, c, d, e, f, g F6, A6L) [1]. |
| 1 | PF03997 | VPS28 | VPS28 protein |  |
| 1 | PF01016 | Ribosomal\_L27 | Ribosomal L27 protein |  |
| 1 | PF10300 | DUF3808 | Protein of unknown function (DUF3808) | This is a family of proteins conserved from fungi to humans. Members of this family also carry a TPR\_2 domain Pfam:PF07719 at their C-terminus. |
| 1 | PF01667 | Ribosomal\_S27e | Ribosomal protein S27 |  |
| 1 | PF01545 | Cation\_efflux | Cation efflux family | Members of this family are integral membrane proteins, that are found to increase tolerance to divalent metal ions such as cadmium, zinc, and cobalt. These proteins are thought to be efflux pumps that remove these ions from cells. |
| 1 | PF00109 | ketoacyl-synt | Beta-ketoacyl synthase, N-terminal domain | The structure of beta-ketoacyl synthase is similar to that of the thiolase family (Pfam:PF00108) and also chalcone synthase. The active site of beta-ketoacyl synthase is located between the N and C-terminal domains. The N-terminal domain contains most of the structures involved in dimer formation and also the active site cysteine [1]. |
| 1 | PF09801 | SYS1 | Integral membrane protein S linking to the trans Golgi network | Members of this family are integral membrane proteins involved in protein trafficking between the late Golgi and endosome. They may also serve as a receptor for ADP-ribosylation factor-related protein 1 (ARFRP1) [1]. Sys1p is a small integral membrane protein with four predicted transmembrane domains that localises to the Trans Golgi network TGN in yeast and human cells [2]. |
| 1 | PF06968 | BATS | Biotin and Thiamin Synthesis associated domain | Biotin synthase (BioB), EC:2.8.1.6 , catalyses the last step of the biotin biosynthetic pathway. The reaction consists in the introduction of a sulphur atom into dethiobiotin. BioB functions as a homodimer [1]. Thiamin synthesis if a complex process involving at least six gene products (ThiFSGH, ThiI and ThiJ). Two of the proteins required for the biosynthesis of the thiazole moiety of thiamine (vitamin B(1)) are ThiG and ThiH (this family) and form a heterodimer[2]. Both of these reactions are thought of involve the binding of co-factors, and both function as dimers [1,2]. This domain therefore may be involved in co-factor binding or dimerisation (Finn, RD personal observation). |
| 1 | PF00365 | PFK | Phosphofructokinase |  |
| 1 | PF03446 | NAD\_binding\_2 | NAD binding domain of 6-phosphogluconate dehydrogenase | The NAD binding domain of 6-phosphogluconate dehydrogenase adopts a Rossmann fold. |
| 1 | PF03162 | Y\_phosphatase2 | Tyrosine phosphatase family | This family is closely related to the Pfam:PF00102 and Pfam:PF00782 families. |
| 1 | PF00328 | His\_Phos\_2 | Histidine phosphatase superfamily (branch 2) | The histidine phosphatase superfamily is so named because catalysis centres on a conserved His residue that is transiently phosphorylated during the catalytic cycle. Other conserved residues contribute to a 'phosphate pocket' and interact with the phospho group of substrate before, during and after its transfer to the His residue. Structure and sequence analyses show that different families contribute different additional residues to the 'phosphate pocket' and, more surprisingly, differ in the position, in sequence and in three dimensions, of a catalytically essential acidic residue. The superfamily may be divided into two main branches.The smaller branch 2 contains predominantly eukaryotic proteins. The catalytic functions in members include phytase, glucose-1-phosphatase and multiple inositol polyphosphate phosphatase. The in vivo roles of the mammalian acid phosphatases in branch 2 are not fully understood, although activity against lysophosphatidic acid and tyrosine-phosphorylated proteins has been demonstrated. |
| 1 | PF11894 | DUF3414 | Protein of unknown function (DUF3414) | This family of proteins are functionally uncharacterised. This protein is found in eukaryotes. Proteins in this family are typically between 764 to 2011 amino acids in length. This protein has a conserved LLG sequence motif. |
| 1 | PF02458 | Transferase | Transferase family | This family includes a number of transferase enzymes. These include anthranilate N-hydroxycinnamoyl/benzoyltransferase that catalyses the first committed reaction of phytoalexin biosynthesis [1]. Deacetylvindoline 4-O-acetyltransferase EC:2.3.1.107 catalyses the last step in vindoline biosynthesis is also a member of this family [2]. The motif HXXXD is probably part of the active site. The family also includes trichothecene 3-O-acetyltransferase. |
| 1 | PF10262 | Rdx | Rdx family | This entry is an approximately 100 residue region of selenoprotein-T, conserved from plants to humans. The protein binds to UDP-glucose:glycoprotein glucosyltransferase (UGTR), the endoplasmic reticulum (ER)-resident protein, which is known to be involved in the quality control of protein folding [1]. Selenium (Se) plays an essential role in cell survival and most of the effects of Se are probably mediated by selenoproteins, including selenoprotein T. However, despite its binding to UGTR and that its mRNA is up-regulated in extended asphyxia, the function of the protein and hence of this region of it is unknown [2]. Selenoprotein W contains selenium as selenocysteine in the primary protein structure and levels of this selenoprotein are affected by selenium [3]. |
| 1 | PF00024 | PAN\_1 | PAN domain | The PAN domain [1] contains a conserved core of three disulphide bridges. In some members of the family there is an additional fourth disulphide bridge the links the N and C termini of the domain. The domain is found in diverse proteins, in some they mediate protein-protein interactions, in others they mediate protein-carbohydrate interactions. |
| 1 | PF00583 | Acetyltransf\_1 | Acetyltransferase (GNAT) family | This family contains proteins with N-acetyltransferase functions such as Elp3-related proteins. |
| 1 | PF11698 | V-ATPase\_H\_C | V-ATPase subunit H | The yeast Saccharomyces cerevisiae vacuolar H+-ATPase (V-ATPase) is a multisubunit complex responsible for acidifying organelles. It functions as an ATP dependent proton pump that transports protons across a lipid bilayer. This domain corresponds to the C terminal domain of the H subunit of V-ATPase. The N-terminal domain is required for the activation of the complex whereas the C-terminal domain is required for coupling ATP hydrolysis to proton translocation [3]. |
| 1 | PF00686 | CBM\_20 | Starch binding domain |  |
| 1 | PF00270 | DEAD | DEAD/DEAH box helicase | Members of this family include the DEAD and DEAH box helicases. Helicases are involved in unwinding nucleic acids. The DEAD box helicases are involved in various aspects of RNA metabolism, including nuclear transcription, pre mRNA splicing, ribosome biogenesis, nucleocytoplasmic transport, translation, RNA decay and organellar gene expression. |
| 1 | PF09350 | DUF1992 | Domain of unknown function (DUF1992) | This family of proteins are functionally uncharacterised. |
| 1 | PF01127 | Sdh\_cyt | Succinate dehydrogenase/Fumarate reductase transmembrane subunit | This family includes a transmembrane protein from both the Succinate dehydrogenase and Fumarate reductase complexes. |
| 1 | PF00248 | Aldo\_ket\_red | Aldo/keto reductase family | This family includes a number of K+ ion channel beta chain regulatory domains - these are reported to have oxidoreductase activity [2]. |
| 1 | PF00717 | Peptidase\_S24 | Peptidase S24-like |  |
| 1 | PF05127 | Helicase\_RecD | Helicase | This domain contains a P-loop (Walker A) motif, suggesting that it has ATPase activity, and a Walker B motif. In tRNA(Met) cytidine acetyltransferase (TmcA) it may function as an RNA helicase motor (driven by ATP hydrolysis) which delivers the wobble base to the active centre of the GCN5-related N-acetyltransferase (GNAT) domain [1]. It is found in the bacterial exodeoxyribonuclease V alpha chain (RecD), which has 5'-3' helicase activity. It is structurally similar to the motor domain 1A in other SF1 helicases [2]. |
| 1 | PF01105 | EMP24\_GP25L | emp24/gp25L/p24 family/GOLD | Members of this family are implicated in bringing cargo forward from the ER and binding to coat proteins by their cytoplasmic domains. This domain corresponds closely to the beta-strand rich GOLD domain described in [2]. The GOLD domain is always found combined with lipid- or membrane-association domains [2]. |
| 1 | PF00152 | tRNA-synt\_2 | tRNA synthetases class II (D, K and N) |  |
| 1 | PF13622 | 4HBT\_3 | Thioesterase-like superfamily | This family contains a wide variety of enzymes, principally thioesterases. These enzymes are part of the Hotdog fold superfamily [1]. |
| 1 | PF05879 | RHD3 | Root hair defective 3 GTP-binding protein (RHD3) | This family consists of several eukaryotic root hair defective 3 like GTP-binding proteins. It has been speculated that the RHD3 protein is a member of a novel class of GTP-binding proteins that is widespread in eukaryotes and required for regulated cell enlargement [1]. The family also contains the homologous yeast synthetic enhancement of YOP1 (SEY1) protein which is involved in membrane trafficking [2]. |
| 1 | PF00731 | AIRC | AIR carboxylase | Members of this family catalyse the decarboxylation of 1-(5-phosphoribosyl)-5-amino-4-imidazole-carboxylate (AIR). This family catalyse the sixth step of de novo purine biosynthesis. Some members of this family contain two copies of this domain. |
| 1 | PF06011 | TRP | Transient receptor potential (TRP) ion channel | This family of proteins are transient receptor potential (TRP) ion channels. They are essential for cellular viability and are involved in cell growth and cell wall synthesis [1]. The genes for these proteins are homologous to polycystic kidney disease related ion channel genes [1]. |
| 1 | PF12774 | AAA\_6 | Hydrolytic ATP binding site of dynein motor region D1 | the 380 kDa motor unit of dynein belongs to the AAA class of chaperone-like ATPases. The core of the 380 kDa motor unit contains a concatenated chain of six AAA modules, of which four correspond to the ATP binding sites with P-loop signatures described previously, and two are modules in which the P loop has been lost in evolution. This particular family is the D1 unit of the motor and contains the hydrolytic ATP binding site [1]. |
| 1 | PF00488 | MutS\_V | MutS domain V | This domain is found in proteins of the MutS family (DNA mismatch repair proteins) and is found associated with Pfam:PF01624, Pfam:PF05188, Pfam:PF05192 and Pfam:PF05190. The mutS family of proteins is named after the Salmonella typhimurium MutS protein involved in mismatch repair; other members of the family included the eukaryotic MSH 1,2,3, 4,5 and 6 proteins. These have various roles in DNA repair and recombination. Human MSH has been implicated in non-polyposis colorectal carcinoma (HNPCC) and is a mismatch binding protein [2]. The aligned region corresponds with domain V of Thermus aquaticus MutS as characterised in [4], which contains a Walker A motif, and is structurally similar to the ATPase domain of ABC transporters. |
| 1 | PF09769 | ApoO | Apolipoprotein O | Members of this family promote cholesterol efflux from macrophage cells. They are present in various lipoprotein complexes, including HDL, LDL and VLDL. The apoprotein is secreted by a microsomal triglyceride transfer protein (MTTP)-dependent mechanism, probably as a VLDL-associated protein that is subsequently transferred to HDL [1]. |
| 1 | PF01813 | ATP-synt\_D | ATP synthase subunit D | This is a family of subunit D form various ATP synthases including V-type H+ transporting and Na+ dependent. Subunit D is suggested to be an integral part of the catalytic sector of the V-ATPase [2]. |
| 1 | PF03914 | CBF | CBF/Mak21 family |  |
| 1 | PF00368 | HMG-CoA\_red | Hydroxymethylglutaryl-coenzyme A reductase | The HMG-CoA reductases catalyse the conversion of HMG-CoA to mevalonate, which is the rate-limiting step in the synthesis of isoprenoids like cholesterol. Probably because of the critical role of this enzyme in cholesterol homeostasis, mammalian HMG-CoA reductase is heavily regulated at the transcriptional, translational, and post-translational levels [2]. |
| 1 | PF01148 | CTP\_transf\_1 | Cytidylyltransferase family | The members of this family are integral membrane protein cytidylyltransferases. The family includes phosphatidate cytidylyltransferase EC:2.7.7.41 as well as Sec59 from yeast. Sec59 is a dolichol kinase EC:2.7.1.108. |
| 1 | PF13508 | Acetyltransf\_7 | Acetyltransferase (GNAT) domain | This domain catalyses N-acetyltransferase reactions. |
| 1 | PF00141 | peroxidase | Peroxidase |  |
| 1 | PF00303 | Thymidylat\_synt | Thymidylate synthase | Swiss:P28176 is not included as a member of this family, Although annotated as such there is no significant sequence similarity to other members. |
| 1 | PF13414 | TPR\_11 | TPR repeat |  |
| 1 | PF06280 | DUF1034 | Fn3-like domain (DUF1034) | This family consists of several domains of unknown function which are present in several bacterial and plant peptidases. This domain is found in conjunction with Pfam:PF00082, Pfam:PF02225 and is often found with Pfam:PF00746. This domain has a structure similar to an Fn3 domain [1]. |
| 1 | PF05916 | Sld5 | GINS complex protein | The eukaryotic GINS complex is essential for the initiation and elongation phases of DNA replication [1-3]. It consists of four paralogous protein subunits (Sld5, Psf1, Psf2 and Psf3), all of which are included in this family. The GINS complex is conserved from yeast to humans, and has been shown in human to bind directly to DNA primase [4]. |
| 1 | PF01679 | Pmp3 | Proteolipid membrane potential modulator | Pmp3 is an evolutionarily conserved proteolipid in the plasma membrane which, in S. pombe, is transcriptionally regulated by the Spc1 stress MAPK (mitogen-activated protein kinases) pathway. It functions to modulate the membrane potential, particularly to resist high cellular cation concentration. In eukaryotic organisms, stress-activated mitogen-activated protein kinases play crucial roles in transmitting environmental signals that will regulate gene expression for allowing the cell to adapt to cellular stress. Pmp3-like proteins are highly conserved in bacteria, yeast, nematode and plants. |
| 1 | PF12838 | Fer4\_7 | 4Fe-4S dicluster domain | Superfamily includes proteins containing domains which bind to iron-sulfur clusters. Members include bacterial ferredoxins, various dehydrogenases, and various reductases. Structure of the domain is an alpha-antiparallel beta sandwich. Domain contains two 4Fe4S clusters. |
| 1 | PF03271 | EB1 | EB1-like C-terminal motif | This motif is found at the C-terminus of proteins that are related to the EB1 protein. The EB1 proteins contain an N-terminal CH domain Pfam:PF00307. The human EB1 protein was originally discovered as a protein interacting with the C-terminus of the APC protein. This interaction is often disrupted in colon cancer, due to deletions affecting the APC C-terminus. Several EB1 orthologues are also included in this family. The interaction between EB1 and APC has been shown to have a potent synergistic effect on microtubule polymerisation. Neither of EB1 or APC alone has this effect. It is thought that EB1 targets APC to the + ends of microtubules, where APC promotes microtubule polymerisation. This process is regulated by APC phosphorylation by Cdc2, which disrupts APC-EB1 binding. Human EB1 protein can functionally substitute for the yeast EB1 homologue Mal3. In addition, Mal3 can substitute for human EB1 in promoting microtubule polymerisation with APC. |
| 1 | PF01261 | AP\_endonuc\_2 | Xylose isomerase-like TIM barrel | This TIM alpha/beta barrel structure is found in xylose isomerase (Swiss:P19148) and in endonuclease IV (Swiss:P12638, EC:3.1.21.2). This domain is also found in the N termini of bacterial myo-inositol catabolism proteins. These are involved in the myo-inositol catabolism pathway, and is required for growth on myo-inositol in Rhizobium leguminosarum bv. viciae [1]. |
| 1 | PF10288 | DUF2392 | Protein of unknown function (DUF2392) | This is a family of proteins conserved from plants to humans. The function is not known. It carries a characteristic GRG sequence motif. |
| 1 | PF06089 | Asparaginase\_II | L-asparaginase II | This family consists of several bacterial L-asparaginase II proteins. L-asparaginase (EC:3.5.1.1) catalyses the hydrolysis of L-asparagine to L-aspartate and ammonium. Rhizobium etli possesses two asparaginases: asparaginase I, which is thermostable and constitutive, and asparaginase II, which is thermolabile, induced by asparagine and repressed by the carbon source [1]. |
| 1 | PF03198 | Glyco\_hydro\_72 | Glucanosyltransferase | This is a family of glycosylphosphatidylinositol-anchored beta(1-3)glucanosyltransferases. The active site residues in the Aspergillus fumigatus example Swiss:B0XT72 are the two glutamate residues at 160 and 261 [3]. |
| 1 | PF03949 | Malic\_M | Malic enzyme, NAD binding domain |  |
| 1 | PF09370 | TIM-br\_sig\_trns | TIM-barrel signal transduction protein | This domain is likely to have a TIM barrel fold related to IGPS. Although this family of proteins are functionally uncharacterised this domain is found as an N-terminal domain of sigma 54 -dependent transcriptional activators (enhancer-binding proteins) suggesting a potential role in signal recognition/receiving and signal transduction. |
| 1 | PF13637 | Ank\_4 | Ankyrin repeats (many copies) |  |
| 1 | PF04408 | HA2 | Helicase associated domain (HA2) | This presumed domain is about 90 amino acid residues in length. It is found is a diverse set of RNA helicases. Its function is unknown, however it seems likely to be involved in nucleic acid binding. |
| 1 | PF02698 | DUF218 | DUF218 domain | This large family of proteins contains several highly conserved charged amino acids, suggesting this may be an enzymatic domain (Bateman A pers. obs). The family includes SanA Swiss:P33017 that is involved in Vancomycin resistance [1]. This protein may be involved in murein synthesis [2]. |
| 1 | PF01088 | Peptidase\_C12 | Ubiquitin carboxyl-terminal hydrolase, family 1 |  |
| 1 | PF06042 | DUF925 | Bacterial protein of unknown function (DUF925) | This family consists of several hypothetical bacterial proteins of unknown function. This family was recently identified as belonging to the nucleotidyltransferase superfamily [1]. |
| 1 | PF07717 | OB\_NTP\_bind | Oligonucleotide/oligosaccharide-binding (OB)-fold | This family is found towards the C-terminus of the DEAD-box helicases (Pfam:PF00270). In these helicases it is apparently always found in association with Pfam:PF04408. There do seem to be a couple of instances where it occurs by itself - e.g. Swiss:Q84VZ2. The structure PDB:3i4u adopts an OB-fold. helicases (Pfam:PF00270). In these helicases it is apparently always found in association with Pfam:PF04408. This C-terminal domain of the yeast helicase contains an oligonucleotide/oligosaccharide-binding (OB)-fold which seems to be placed at the entrance of the putative nucleic acid cavity. It also constitutes the binding site for the G-patch-containing domain of Pfa1p. When found on DEAH/RHA helicases, this domain is central to the regulation of the helicase activity through its binding of both RNA and G-patch domain proteins [1]. |
| 1 | PF09797 | NatB\_MDM20 | N-acetyltransferase B complex (NatB) non catalytic subunit | This is the non-catalytic subunit of the N-terminal acetyltransferase B complex (NatB). The NatB complex catalyses the acetylation of the amino-terminal methionine residue of all proteins beginning with Met-Asp or Met-Glu and of some proteins beginning with Met-Asn or Met-Met. In Saccharomyces cerevisiae this subunit is called MDM20 and in Schizosaccharomyces pombe it is called Arm1. NatB acetylates the Tpm1 protein and regulates and tropomyocin-actin interactions. This subunit is required by the NatB complex for the N-terminal acetylation of Tpm1 [1]. |
| 1 | PF12751 | Vac7 | Vacuolar segregation subunit 7 | Vac7 is localised at the vacuole membrane, a location which is consistent with its involvement in vacuole morphology and inheritance [1]. Vac7 has been shown to function as an upstream regulator of the Fab1 lipid kinase pathway [2]. The Fab1 lipid p[pathway is important for correct regulation of membrane trafficking events. |
| 1 | PF01793 | Glyco\_transf\_15 | Glycolipid 2-alpha-mannosyltransferase | This is a family of alpha-1,2 mannosyl-transferases involved in N-linked and O-linked glycosylation of proteins. Some of the enzymes in this family have been shown to be involved in O- and N-linked glycan modifications in the Golgi [1]. |
| 1 | PF05255 | UPF0220 | Uncharacterised protein family (UPF0220) | This family of proteins is functionally uncharacterised. |
| 1 | PF00561 | Abhydrolase\_1 | alpha/beta hydrolase fold | This catalytic domain is found in a very wide range of enzymes. |
| 1 | PF08610 | Pex16 | Peroxisomal membrane protein (Pex16) | Pex16 is a peripheral protein located at the matrix face of the peroxisomal membrane [1]. |
| 1 | PF02441 | Flavoprotein | Flavoprotein | This family contains diverse flavoprotein enzymes. This family includes epidermin biosynthesis protein, EpiD Swiss:P30197, which has been shown to be a flavoprotein that binds FMN [1]. This enzyme catalyses the removal of two reducing equivalents from the cysteine residue of the C-terminal meso-lanthionine of epidermin to form a --C==C-- double bond. This family also includes the B chain of dipicolinate synthase a small polar molecule that accumulates to high concentrations in bacterial endospores, and is thought to play a role in spore heat resistance, or the maintenance of heat resistance [2]. dipicolinate synthase catalyses the formation of dipicolinic acid from dihydroxydipicolinic acid. This family also includes phenyl-acrylic acid decarboxylase Swiss:P33751 (EC:4.1.1.-) [3]. |
| 1 | PF04511 | DER1 | Der1-like family | The endoplasmic reticulum (ER) of the yeast Saccharomyces cerevisiae contains of proteolytic system able to selectively degrade misfolded lumenal secretory proteins. For examination of the components involved in this degradation process, mutants were isolated. They could be divided into four complementation groups. The mutations led to stabilisation of two different substrates for this process. The mutant classes were called 'der' for 'degradation in the ER'. DER1 was cloned by complementation of the der1-2 mutation. The DER1 gene codes for a novel, hydrophobic protein, that is localised to the ER. Deletion of DER1 abolished degradation of the substrate proteins. The function of the Der1 protein seems to be specifically required for the degradation process associated with the ER [1]. Interestingly this family seems distantly related to the Rhomboid family of membrane peptidases. Suggesting that this family may also mediate degradation of misfolded proteins (Bateman A pers. obs.). |
| 1 | PF00668 | Condensation | Condensation domain | This domain is found in many multi-domain enzymes which synthesise peptide antibiotics. This domain catalyses a condensation reaction to form peptide bonds in non- ribosomal peptide biosynthesis. It is usually found to the carboxy side of a phosphopantetheine binding domain (Pfam:PF00550). It has been shown that mutations in the HHXXXDG motif abolish activity suggesting this is part of the active site [1]. |
| 1 | PF02353 | CMAS | Mycolic acid cyclopropane synthetase | This family consist of Cyclopropane-fatty-acyl-phospholipid synthase or CFA synthase EC:2.1.1.79 this enzyme catalyse the reaction: S-adenosyl-L-methionine + phospholipid olefinic fatty acid <=> S-adenosyl-L-homocysteine + phospholipid cyclopropane fatty acid. |
| 1 | PF10247 | Romo1 | Reactive mitochondrial oxygen species modulator 1 | This is a family of small, approximately 100 amino acid, proteins found from yeasts to humans. The majority of endogenous reactive oxygen species (ROS) in cells are produced by the mitochondrial respiratory chain. An increase or imbalance in ROS alters the intracellular redox homeostasis, triggers DNA damage, and may contribute to cancer development and progression [1]. Members of this family are mitochondrial reactive oxygen species modulator 1 (Romo1) proteins that are responsible for increasing the level of ROS in cells. Increased Romo1 expression can have a number of other effects including: inducing premature senescence of cultured human fibroblasts [2,3] and increased resistance to 5-fluorouracil [4]. |
| 1 | PF01872 | RibD\_C | RibD C-terminal domain | The function of this domain is not known, but it is thought to be involved in riboflavin biosynthesis. This domain is found in the C terminus of RibD/RibG Swiss:P25539, in combination with Pfam:PF00383, as well as in isolation in some archaebacterial proteins Swiss:P95872. This family appears to be related to Pfam:PF00186. |
| 1 | PF09206 | ArabFuran-catal | Alpha-L-arabinofuranosidase B, catalytic | Members of this family, which are present in fungal alpha-L-arabinofuranosidase B, adopt a beta-sandwich fold similar to that of Concanavalin A-like lectins/glucanase. The beta-sandwich fold consists of two anti-parallel beta-sheets with seven and and six strands, respectively. In addition, there are four helices outside of the beta-strands. The beta-sandwich strands are closely packed and curved with a jelly roll topology, creating a small catalytic pocket. The domain catalyses the hydrolysis of alpha-1,2-, alpha-1,3- and alpha-1,5-L-arabinofuranosidic bonds in L-arabinose-containing hemicelluloses such as arabinoxylan and L-arabinan [1]. |
| 1 | PF00722 | Glyco\_hydro\_16 | Glycosyl hydrolases family 16 |  |
| 1 | PF04080 | Per1 | Per1-like | PER1 is required for GPI-phospholipase A2 activity and is involved in lipid remodelling of GPI-anchored proteins [2]. |
| 1 | PF03901 | Glyco\_transf\_22 | Alg9-like mannosyltransferase family | Members of this family are mannosyltransferase enzymes [1-2]. At least some members are localised in endoplasmic reticulum and involved in GPI anchor biosynthesis [3-4]. |
| 1 | PF09258 | Glyco\_transf\_64 | Glycosyl transferase family 64 domain | Members of this family catalyse the transfer reaction of N-acetylglucosamine and N-acetylgalactosamine from the respective UDP-sugars to the non-reducing end of [glucuronic acid]beta 1-3[galactose]beta 1-O-naphthalenemethanol, an acceptor substrate analog of the natural common linker of various glycosylaminoglycans. They are also required for the biosynthesis of heparan-sulphate [1]. |
| 1 | PF04909 | Amidohydro\_2 | Amidohydrolase | These proteins are amidohydrolases that are related to Pfam:PF01979 [1]. |
| 1 | PF12695 | Abhydrolase\_5 | Alpha/beta hydrolase family | This family contains a diverse range of alpha/beta hydrolase enzymes. |
| 1 | PF03982 | DAGAT | Diacylglycerol acyltransferase | The terminal step of triacylglycerol (TAG) formation is catalysed by the enzyme diacylglycerol acyltransferase (DAGAT) [1,2]. |
| 1 | PF04926 | PAP\_RNA-bind | Poly(A) polymerase predicted RNA binding domain | Based on its similarity structurally to the RNA recognition motif this domain is thought to be RNA binding [1]. |
| 1 | PF00459 | Inositol\_P | Inositol monophosphatase family |  |
| 1 | PF00840 | Glyco\_hydro\_7 | Glycosyl hydrolase family 7 |  |
| 1 | PF03095 | PTPA | Phosphotyrosyl phosphate activator (PTPA) protein | Phosphotyrosyl phosphatase activator (PTPA) proteins stimulate the phosphotyrosyl phosphatase (PTPase) activity of the dimeric form of protein phosphatase 2A (PP2A). PTPase activity in PP2A (in vitro) is relatively low when compared to the better recognised phosphoserine/ threonine protein phosphorylase activity. The specific biological role of PTPA is unknown, Basal expression of PTPA depends on the activity of a ubiquitous transcription factor, Yin Yang 1 (YY1). The tumour suppressor protein p53 can inhibit PTPA expression through an unknown mechanism that negatively controls YY1 [1]. |
| 1 | PF00930 | DPPIV\_N | Dipeptidyl peptidase IV (DPP IV) N-terminal region | This family is an alignment of the region to the N-terminal side of the active site. The Prosite motif does not correspond to this Pfam entry. |
| 1 | PF00135 | COesterase | Carboxylesterase family |  |
| 1 | PF13185 | GAF\_2 | GAF domain |  |
| 1 | PF00733 | Asn\_synthase | Asparagine synthase | This family is always found associated with Pfam:PF00310. Members of this family catalyse the conversion of aspartate to asparagine. |
| 1 | PF08030 | NAD\_binding\_6 | Ferric reductase NAD binding domain |  |
| 1 | PF03224 | V-ATPase\_H\_N | V-ATPase subunit H | The yeast Saccharomyces cerevisiae vacuolar H+-ATPase (V-ATPase) is a multisubunit complex responsible for acidifying organelles. It functions as an ATP dependent proton pump that transports protons across a lipid bilayer. This domain corresponds to the N terminal domain of the H subunit of V-ATPase. The N-terminal domain is required for the activation of the complex whereas the C-terminal domain is required for coupling ATP hydrolysis to proton translocation [3]. |
| 1 | PF02466 | Tim17 | Tim17/Tim22/Tim23/Pmp24 family | The pre-protein translocase of the mitochondrial outer membrane (Tom) allows the import of pre-proteins from the cytoplasm. Tom forms a complex with a number of proteins, including Tim17. Tim17 and Tim23 are thought to form the translocation channel of the inner membrane. This family includes Tim17, Tim22 and Tim23. This family also includes Pmp24 a peroxisomal protein. The involvement of this domain in the targeting of PMP24 remains to be proved. PMP24 was known as Pmp27 in [3]. |
| 1 | PF13558 | SbcCD\_C | Putative exonuclease SbcCD, C subunit | Possible exonuclease SbcCD, C subunit, on AAA proteins. |
| 1 | PF02516 | STT3 | Oligosaccharyl transferase STT3 subunit | This family consists of the oligosaccharyl transferase STT3 subunit and related proteins. The STT3 subunit is part of the oligosaccharyl transferase (OTase) complex of proteins and is required for its activity [2]. In eukaryotes, OTase transfers a lipid-linked core-oligosaccharide to selected asparagine residues in the ER [2]. In the archaea STT3 occurs alone, rather than in an OTase complex, and is required for N-glycosylation of asparagines [3-4]. |
| 1 | PF04253 | TFR\_dimer | Transferrin receptor-like dimerisation domain | This domain is involved in dimerisation of the transferrin receptor as shown in its crystal structure. |
| 1 | PF03171 | 2OG-FeII\_Oxy | 2OG-Fe(II) oxygenase superfamily | This family contains members of the 2-oxoglutarate (2OG) and Fe(II)-dependent oxygenase superfamily [1]. This family includes the C-terminal of prolyl 4-hydroxylase alpha subunit. The holoenzyme has the activity EC:1.14.11.2 catalysing the reaction: Procollagen L-proline + 2-oxoglutarate + O2 <=> procollagen trans- 4-hydroxy-L-proline + succinate + CO2. The full enzyme consists of a alpha2 beta2 complex with the alpha subunit contributing most of the parts of the active site [3]. The family also includes lysyl hydrolases, isopenicillin synthases and AlkB. |
