# Supplementary material for: Investigating the beneficial traits of Trichoderma hamatum GD12 for sustainable agriculture—insights from genomics
Source: Front Plant Sci. 2013 Jul 30;4:258. doi: 10.3389/fpls.2013.00258 (PMC3726867; doi:10.3389/fpls.2013.00258)
Supplement: Supplementary File S1 — GD12.secretome.no-TMs.faa.pfamscan.html. Frequencies of Pfam domains in predicted secreted proteins encoded in the GD12 genome. [file DataSheet1.ZIP › Supplemental material/Supp6_GD12.secretome.no-TMs.SSCRPs.faa.pfamscan.html]

|  |  |  |  |  |
| --- | --- | --- | --- | --- |
| 3 | PF11951 | Fungal\_trans\_2 | Fungal specific transcription factor domain | This family of are likely to be transcription factors. This protein is found in fungi. Proteins in this family are typically between 454 to 826 amino acids in length. This protein is found associated with Pfam:PF00172. |
| 3 | PF04082 | Fungal\_trans | Fungal specific transcription factor domain |  |
| 2 | PF03313 | SDH\_alpha | Serine dehydratase alpha chain | L-serine dehydratase (EC:4.2.1.13) is a found as a heterodimer of alpha and beta chain or as a fusion of the two chains in a single protein. This enzyme catalyses the deamination of serine to form pyruvate. This enzyme is part of the gluconeogenesis pathway. |
| 2 | PF06766 | Hydrophobin\_2 | Fungal hydrophobin | This is a family of fungal hydrophobins that seems to be restricted to ascomycetes. These are small, moderately hydrophobic extracellular proteins that have eight cysteine residues arranged in a strictly conserved motif. Hydrophobins are generally found on the outer surface of conidia and of the hyphal wall, and may be involved in mediating contact and communication between the fungus and its environment [1]. Note that some family members contain multiple copies. |
| 2 | PF00501 | AMP-binding | AMP-binding enzyme |  |
| 2 | PF00654 | Voltage\_CLC | Voltage gated chloride channel | This family of ion channels contains 10 or 12 transmembrane helices. Each protein forms a single pore. It has been shown that some members of this family form homodimers. In terms of primary structure, they are unrelated to known cation channels or other types of anion channels. Three ClC subfamilies are found in animals. ClC-1 (Swiss:P35523) is involved in setting and restoring the resting membrane potential of skeletal muscle, while other channels play important parts in solute concentration mechanisms in the kidney [3]. These proteins contain two Pfam:PF00571 domains. |
| 2 | PF00734 | CBM\_1 | Fungal cellulose binding domain |  |
| 1 | PF08386 | Abhydrolase\_4 | TAP-like protein | This is a family of putative bacterial peptidases and hydrolases that bear similarity to a tripeptidyl aminopeptidase isolated from Streptomyces lividans (Swiss:Q54410). A member of this family (Swiss:Q6E3K7) is thought to be involved in the C-terminal processing of propionicin F, a bacteriocidin characterised from Propionibacterium freudenreichii [1]. |
| 1 | PF12774 | AAA\_6 | Hydrolytic ATP binding site of dynein motor region D1 | the 380 kDa motor unit of dynein belongs to the AAA class of chaperone-like ATPases. The core of the 380 kDa motor unit contains a concatenated chain of six AAA modules, of which four correspond to the ATP binding sites with P-loop signatures described previously, and two are modules in which the P loop has been lost in evolution. This particular family is the D1 unit of the motor and contains the hydrolytic ATP binding site [1]. |
| 1 | PF10233 | Cg6151-P | Uncharacterized conserved protein CG6151-P | This is a family of small, less than 200 residue long, proteins which are named as CG6151-P proteins that are conserved from fungi to humans. The function is unknown. The fungal members have a characteristic ICP sequence motif. Some members are annotated as putative clathrin-coated vesicle protein but this could not be defined. |
| 1 | PF01058 | Oxidored\_q6 | NADH ubiquinone oxidoreductase, 20 Kd subunit |  |
| 1 | PF12739 | TRAPPC-Trs85 | ER-Golgi trafficking TRAPP I complex 85 kDa subunit | This family is one of the subunits of the TRAPP Golgi trafficking complex. TRAPP subunits are found in two different sized complexes, TRAPP I and TRAPP II, and this Trs85 is in the smaller complex. TRAPP I, but Not TRAPP II, functions in ER-Golgi transport [1]. Trs85p was reported to function in the cytosol-to-vacuole targeting pathway, suggesting a role for this subunit in autophagy as well as in secretion [2]. The overall architecture of TRAPP I shows the other components to be Bet3p (TRAPPC3), Bet5p (TRAPPC1), Trs20p (TRAPPC2) , Trs23p (TRAPPC4), Trs31p (TRAPPC5), Trs33p (TRAPPC6a and b) and Trs85p. |
| 1 | PF11707 | Npa1 | Ribosome 60S biogenesis N-terminal | Npa1p is required for ribosome biogenesis and operates in the same functional environment as Rsa3p and Dbp6p during early maturation of 60S ribosomal subunits [1]. The protein partners of Npa1p include eight putative helicases as well as the novel Npa2p factor. Npa1p can also associate with a subset of H/ACA and C/D small nucleolar RNPs (snoRNPs) involved in the chemical modification of residues in the vicinity of the peptidyl transferase centre [2]. The protein has also been referred to as Urb1, and this domain at the N-terminal is one of several conserved regions along the length. |
| 1 | PF13350 | Y\_phosphatase3 | Tyrosine phosphatase family | This family is closely related to the Pfam:PF00102 and Pfam:PF00782 families. |
| 1 | PF00383 | dCMP\_cyt\_deam\_1 | Cytidine and deoxycytidylate deaminase zinc-binding region |  |
| 1 | PF13639 | zf-RING\_2 | Ring finger domain |  |
| 1 | PF03481 | SUA5 | Putative GTP-binding controlling metal-binding | Structural investigation of this domain suggests that it might be a GTP-binding region that regulates metal binding and involves hydrolysis of ATP to AMP. It is found to the C-terminus of Pfam:PF01300. |
| 1 | PF00175 | NAD\_binding\_1 | Oxidoreductase NAD-binding domain | Xanthine dehydrogenases, that also bind FAD/NAD, have essentially no similarity. |
| 1 | PF12838 | Fer4\_7 | 4Fe-4S dicluster domain | Superfamily includes proteins containing domains which bind to iron-sulfur clusters. Members include bacterial ferredoxins, various dehydrogenases, and various reductases. Structure of the domain is an alpha-antiparallel beta sandwich. Domain contains two 4Fe4S clusters. |
| 1 | PF02574 | S-methyl\_trans | Homocysteine S-methyltransferase | This is a family of related homocysteine S-methyltransferases enzymes: 5-methyltetrahydrofolate--homocysteine S-methyltransferases also known EC:2.1.1.13, [2]; Betaine--homocysteine S-methyltransferase (vitamin B12 dependent), EC:2.1.1.5, [3]; and Homocysteine S-methyltransferase, EC:2.1.1.10, [1]. |
| 1 | PF10288 | DUF2392 | Protein of unknown function (DUF2392) | This is a family of proteins conserved from plants to humans. The function is not known. It carries a characteristic GRG sequence motif. |
| 1 | PF01485 | IBR | IBR domain | The IBR (In Between Ring fingers) domain is often found to occur between pairs of ring fingers (Pfam:PF00097). This domain has also been called the C6HC domain and DRIL (for double RING finger linked) domain [2]. Proteins that contain two Ring fingers and an IBR domain (these proteins are also termed RBR family proteins) are thought to exist in all eukaryotic organisms. RBR family members play roles in protein quality control and can indirectly regulate transcription [3]. Evidence suggests that RBR proteins are often parts of cullin-containing ubiquitin ligase complexes. The ubiquitin ligase Parkin is an RBR family protein whose mutations are involved in forms of familial Parkinson's disease [3][4]. |
| 1 | PF13893 | RRM\_5 | RNA recognition motif. (a.k.a. RRM, RBD, or RNP domain) | The RRM motif is probably diagnostic of an RNA binding protein. RRMs are found in a variety of RNA binding proteins, including various hnRNP proteins, proteins implicated in regulation of alternative splicing, and protein components of snRNPs. The motif also appears in a few single stranded DNA binding proteins. |
| 1 | PF01507 | PAPS\_reduct | Phosphoadenosine phosphosulfate reductase family | This domain is found in phosphoadenosine phosphosulfate (PAPS) reductase enzymes or PAPS sulfotransferase. PAPS reductase is part of the adenine nucleotide alpha hydrolases superfamily also including N type ATP PPases and ATP sulphurylases [1]. The enzyme uses thioredoxin as an electron donor for the reduction of PAPS to phospho-adenosine-phosphate (PAP) [1,2]. It is also found in NodP nodulation protein P from Rhizobium which has ATP sulfurylase activity (sulfate adenylate transferase) [3]. |
| 1 | PF01464 | SLT | Transglycosylase SLT domain | This family is distantly related to Pfam:PF00062. Members are found in phages, type II, type III and type IV secretion systems (reviewed in [4]). |
| 1 | PF00107 | ADH\_zinc\_N | Zinc-binding dehydrogenase |  |
| 1 | PF00293 | NUDIX | NUDIX domain |  |
| 1 | PF00561 | Abhydrolase\_1 | alpha/beta hydrolase fold | This catalytic domain is found in a very wide range of enzymes. |
| 1 | PF07217 | Het-C | Heterokaryon incompatibility protein Het-C | In filamentous fungi, het loci (for heterokaryon incompatibility) are believed to regulate self/nonself-recognition during vegetative growth. As filamentous fungi grow, hyphal fusion occurs within an individual colony to form a network. Hyphal fusion can occur also between different individuals to form a heterokaryon, in which genetically distinct nuclei occupy a common cytoplasm. However, heterokaryotic cells are viable only if the individuals involved have identical alleles at all het loci [1]. |
| 1 | PF01667 | Ribosomal\_S27e | Ribosomal protein S27 |  |
| 1 | PF14295 | PAN\_4 | PAN domain |  |
| 1 | PF11635 | Med16 | Mediator complex subunit 16 | Mediator is a large complex of up to 33 proteins that is conserved from plants through fungi to humans - the number and representation of individual subunits varying with species [1-2]. It is arranged into four different sections, a core, a head, a tail and a kinase-activity part, and the number of subunits within each of these is what varies with species. Overall, Mediator regulates the transcriptional activity of RNA polymerase II but it would appear that each of the four different sections has a slightly different function. Med16 is one of the subunits of the Tail portion of the Mediator complex and is required for lipopolysaccharide gene-expression [4]. Several members including the human protein, Swiss:Q9Y2X0, have one or more WD40 domains on them, Pfam:PF00400. |
| 1 | PF00069 | Pkinase | Protein kinase domain |  |
| 1 | PF12417 | DUF3669 | Zinc finger protein | This domain family is found in eukaryotes, and is typically between 64 and 80 amino acids in length. |
| 1 | PF01470 | Peptidase\_C15 | Pyroglutamyl peptidase |  |
| 1 | PF00378 | ECH | Enoyl-CoA hydratase/isomerase family | This family contains a diverse set of enzymes including: Enoyl-CoA hydratase (Swiss:Q13011). Napthoate synthase (Swiss:P27290). Carnitate racemase (Swiss:P31551). 3-hydoxybutyryl-CoA dehydratase (Swiss:P52046). Dodecanoyl-CoA delta-isomerase (Swiss:P42126). |
| 1 | PF03446 | NAD\_binding\_2 | NAD binding domain of 6-phosphogluconate dehydrogenase | The NAD binding domain of 6-phosphogluconate dehydrogenase adopts a Rossmann fold. |
| 1 | PF01048 | PNP\_UDP\_1 | Phosphorylase superfamily | Members of this family include: purine nucleoside phosphorylase (PNP) Uridine phosphorylase (UdRPase) 5'-methylthioadenosine phosphorylase (MTA phosphorylase) |
| 1 | PF01300 | Sua5\_yciO\_yrdC | Telomere recombination | This domain has been shown to bind preferentially to dsRNA [1]. The domain is found in SUA5 Swiss:P32579 as well as HypF and YrdC Swiss:P45748. It has also been shown to be required for telomere recombniation in yeast. |
| 1 | PF13561 | adh\_short\_C2 | Enoyl-(Acyl carrier protein) reductase |  |
| 1 | PF10294 | Methyltransf\_16 | Putative methyltransferase |  |
| 1 | PF09206 | ArabFuran-catal | Alpha-L-arabinofuranosidase B, catalytic | Members of this family, which are present in fungal alpha-L-arabinofuranosidase B, adopt a beta-sandwich fold similar to that of Concanavalin A-like lectins/glucanase. The beta-sandwich fold consists of two anti-parallel beta-sheets with seven and and six strands, respectively. In addition, there are four helices outside of the beta-strands. The beta-sandwich strands are closely packed and curved with a jelly roll topology, creating a small catalytic pocket. The domain catalyses the hydrolysis of alpha-1,2-, alpha-1,3- and alpha-1,5-L-arabinofuranosidic bonds in L-arabinose-containing hemicelluloses such as arabinoxylan and L-arabinan [1]. |
| 1 | PF02815 | MIR | MIR domain | The MIR (protein mannosyltransferase, IP3R and RyR) domain is a domain that may have a ligand transferase function [1]. |
| 1 | PF03901 | Glyco\_transf\_22 | Alg9-like mannosyltransferase family | Members of this family are mannosyltransferase enzymes [1-2]. At least some members are localised in endoplasmic reticulum and involved in GPI anchor biosynthesis [3-4]. |
| 1 | PF00840 | Glyco\_hydro\_7 | Glycosyl hydrolase family 7 |  |
| 1 | PF13185 | GAF\_2 | GAF domain |  |
| 1 | PF08030 | NAD\_binding\_6 | Ferric reductase NAD binding domain |  |
| 1 | PF00122 | E1-E2\_ATPase | E1-E2 ATPase |  |
| 1 | PF13558 | SbcCD\_C | Putative exonuclease SbcCD, C subunit | Possible exonuclease SbcCD, C subunit, on AAA proteins. |
| 1 | PF00171 | Aldedh | Aldehyde dehydrogenase family | This family of dehydrogenases act on aldehyde substrates. Members use NADP as a cofactor. The family includes the following members: The prototypical members are the aldehyde dehydrogenases Swiss:P00352 EC:1.2.1.3. Succinate-semialdehyde dehydrogenase Swiss:P25526 EC:1.2.1.16. Lactaldehyde dehydrogenase Swiss:P25553 EC:1.2.1.22. Benzaldehyde dehydrogenase Swiss:P43503 EC:1.2.1.28. Methylmalonate-semialdehyde dehydrogenase Swiss:Q02252 EC:1.2.1.27. Glyceraldehyde-3-phosphate dehydrogenase Swiss:P81406 EC:1.2.1.9. Delta-1-pyrroline-5-carboxylate dehydrogenase Swiss:P30038 EC: 1.5.1.12. Acetaldehyde dehydrogenase Swiss:P17547 EC:1.2.1.10. Glutamate-5-semialdehyde dehydrogenase Swiss:P07004 EC:1.2.1.41. This family also includes omega crystallin Swiss:P30842 an eye lens protein from squid and octopus that has little aldehyde dehydrogenase activity. |
| 1 | PF02585 | PIG-L | GlcNAc-PI de-N-acetylase | Members of this family are related to PIG-L an N-acetylglucosaminylphosphatidylinositol de-N-acetylase (EC:3.5.1.89) that catalyses the second step in GPI biosynthesis [1]. |
| 1 | PF04828 | GFA | Glutathione-dependent formaldehyde-activating enzyme |  |
| 1 | PF00076 | RRM\_1 | RNA recognition motif. (a.k.a. RRM, RBD, or RNP domain) | The RRM motif is probably diagnostic of an RNA binding protein. RRMs are found in a variety of RNA binding proteins, including various hnRNP proteins, proteins implicated in regulation of alternative splicing, and protein components of snRNPs. The motif also appears in a few single stranded DNA binding proteins. The RRM structure consists of four strands and two helices arranged in an alpha/beta sandwich, with a third helix present during RNA binding in some cases The C-terminal beta strand (4th strand) and final helix are hard to align and have been omitted in the SEED alignment The LA proteins (Swiss:P05455) have an N terminal rrm which is included in the seed. There is a second region towards the C terminus that has some features characteristic of a rrm but does not appear to have the important structural core of a rrm. The LA proteins (Swiss:P05455) are one of the main autoantigens in Systemic lupus erythematosus (SLE), an autoimmune disease. |
| 1 | PF00152 | tRNA-synt\_2 | tRNA synthetases class II (D, K and N) |  |
